# Transitioning from MODIS to VIIRS Global Water Reservoir Product

**DOI:** 10.1038/s41597-024-03028-2

**Published:** 2024-02-15

**Authors:** Deep Shah, Shuai Zhang, Sudipta Sarkar, Carol Davidson, Rui Zhang, Maosheng Zhao, Sadashiva Devadiga, Praveen Noojipady, Miguel O. Román, Huilin Gao

**Affiliations:** 1 Zachry Department of Civil and Environmental Engineering, Texas A&M University, College Station, TX USA; 2 Science Systems and Applications Inc., Lanham, MD USA; 3 Terrestrial Information Systems Laboratory, NASA Goddard Space Flight Center, Greenbelt, MD USA; 4 Global Science & Technology Inc., Greenbelt, MD USA; 5 Leidos, Inc., Reston, VA USA

**Keywords:** Climate sciences, Hydrology

## Abstract

Reservoirs play a crucial role in regulating water availability and enhancing water security. Here, we develop NASA’s Visible Infrared Imaging Radiometer Suite (VIIRS) based Global Water Reservoir (GWR) product, consisting of measurements of reservoir area, elevation, storage, evaporation rate, and evaporation loss for 164 large global reservoirs. The dataset is available at 8-day and monthly temporal resolutions. Since the Moderate Resolution Imaging Spectroradiometer (MODIS) is close to the end of its life, we further evaluated the consistency between MODIS and VIIRS-based GWR to ensure continuity to the 20+ year MODIS GWR product. Independent assessment of VIIRS reservoir storage (8-day) retrievals against *in-situ* measurements shows an average of R^2^ = 0.84, RMSE = 0.47 km^3^, and NRMSE = 16.45%. The evaporation rate has an average of R^2^ = 0.56, RMSE = 1.32 mm/day, and NRMSE = 28.14%. Furthermore, results show good consistency (R^2^ ≥ 0.90) between the VIIRS and MODIS-based product components, confirming that long-term data continuity can be achieved. This dataset can provide valuable insights for long-term trend analysis, hydrological modeling, and understanding hydroclimatic extremes in the context of reservoirs.

## Background & Summary

Reservoirs play a critical role in water resource management, altering streamflow variability and providing essential functions such as flood control, hydropower generation, irrigation, and municipal water supply. Although extensive data exist regarding dam attributes and catchment properties^1–5^, long-term and continuous observations of reservoir dynamics (e.g., storage, area, elevation, and evaporation estimates) are predominantly limited to developed countries. Due to the strategic and geopolitical significance of reservoirs within and across nations, records of reservoirs are often not publicly shared. Additionally, access to data is challenging in international river basins, which cover almost 50% of the global land area^6^. Therefore, developing transparent, long-term, publicly accessible global reservoir datasets is crucial for efficient water planning and management across policy-relevant scales, from regional to global.

Satellite remote sensing is a crucial tool for generating continuous, long-term reservoir observations at a global scale. Radar altimeters have been widely used to measure lake and reservoir water levels^7,8^. Notably, publicly available databases such as the Global Reservoir and Lake Monitor (G-REALM^9^), Hydroweb^10^, and the Database for Hydrological Time Series of Inland Waters (DAHITI^11^) are extensively used to monitor water level of lakes and reservoirs. Additionally, estimating global lake/reservoir surface area variations using optical sensors such as Landsat and Moderate Resolution Imaging Spectroradiometer (MODIS) data is prevalent in the field^12–20^. Concurrently, numerous studies have focused on generating satellite-based reservoir storage estimations by integrating elevation and area observations from multiple missions^10,19–24^.

Reservoirs and lakes lose substantial water through evaporation^25–28^. Unlike natural lakes, artificial reservoirs have a more significant trend in evaporation losses^25^. Additionally, the combined effects of population growth and climate change are exacerbating the impact on freshwater availability in reservoirs by reducing storage capacity and increasing evaporation losses^26^. Despite the growing demand for a global reservoir evaporation dataset, very few remotely sensed evaporation datasets are available. Efforts have been made to estimate reservoir evaporation rates and losses by combining modeling and remote sensing approaches^25,27,29–35^. For example, Zhao and Gao^30^ used the Penman equation (with the heat storage and fetch effects addressed) to generate a comprehensive evaporation data record for over 700 reservoirs in the United States. Zhao *et al*.^31^ further improved the estimation by incorporating Terra/Aqua MODIS water surface temperature data to enhance the assessment of the heat storage effect. Some studies^36–38^ have evaluated different evaporation estimation methods at specific locations. For instance, Meng *et al*.^37^ assessed the performances of nine evaporation methods at different timescales and calibrated them using continuous eddy covariance (EC) observation data over Erhai Lake. Moreover, accurate evaporation estimation is essential for reservoirs in arid and semi-arid regions^39^ and at high latitudes^25^. Recent research has demonstrated that integrating evaporation information with storage data can significantly improve the detection and characterization of reservoir-based droughts^40^. In summary, a strong need exists for a global operational reservoir product that simultaneously provides consistent estimates of reservoir elevation, area, storage, evaporation rate, and evaporation losses.

Therefore, we developed National Aeronautics and Space Administration (NASA)‘s long-term standard global water reservoir (GWR) product suite from moderate-resolution remote sensing data such as the MODIS and the Visible Infrared Imaging Radiometer Suite (VIIRS)^41^. The product suite provides 8-day and monthly measurements of area, elevation, and storage, along with monthly evaporation rates and evaporative volumetric losses for 164 global reservoirs. This global reservoir product is operational and is capable of monitoring storage and evaporation simultaneously. While Li *et al*.^41^ introduced the product suite and primarily focused on evaluating the MODIS GWR product, this study aims to introduce the newly released VIIRS GWR standard operational product and establish MODIS/VIIRS product continuity.

Time-series continuity and the seamless transition between products are essential prerequisites for long-term change monitoring, trend identification, and operational applications. Given the forthcoming end-of-life plans for the Terra and Aqua missions, the issue of data continuity requires greater attention, particularly considering the unprecedented usage of MODIS data products (with over 22,000 publications and 350,000 citations: https://modis.gsfc.nasa.gov/sci_team/pubs/). Since VIIRS will remain operational for the next few decades (with the launches of additional Joint Polar Satellite System (JPSS) 3 and 4 satellites scheduled for 2027 and 2032, respectively), it will serve as a viable successor to MODIS observations for ensuring long-term GWR continuity.

Although MODIS and VIIRS are very similar instruments, they have some differences in terms of spectral bands, spatial resolutions, and overpass times^42^. These dissimilarities could potentially translate into disparities within the resultant data products. Hence, NASA’s Land Discipline Team within the Terra, Aqua, and Suomi National Polar-orbiting Partnership (TASNPP) (comprising science team members responsible for each product) is actively working towards achieving the continuity of all global land data products from MODIS to VIIRS^42^. Considerable effort and resources are dedicated to cross-calibration, science product generation, product documentation, and quality assurance to ensure dynamic continuity across research and operational disciplines. For example, VIIRS and MODIS underwent cross-calibration using a global network of benchmarking sites known as BELMANIP2 to align VIIRS raw values with MODIS Aqua, ensuring agreement within the required margin of 1%^42^. The cross-calibration strategy employed three techniques: (1) Cross-calibrating the near-infrared (NIR) VIIRS I2 band and MODIS band 2 at the BELMANIP2 sites^42^; (2) Transferring the VIIRS NIR adjusted calibration to the VIIRS visible bands using data from Deep Convective Clouds (DCC)^42,43^; and (3) Extending the adjusted VIIRS NIR calibration to the VIIRS short wave infrared (SWIR) bands through sun-glint observations^42,44^. Therefore, as part of the extensive efforts led by TASNPP, the evaluation of continuity between NASA’s MODIS and VIIRS operational GWR products holds immense significance.

Recognizing the significant importance of continuity, numerous peer-reviewed studies have evaluated the consistency between MODIS and VIIRS products^45–49^. For instance, Frey *et al*.^45^ examined the continuity of MODIS-VIIRS cloud masks, while Riggs and Hall^47^ established continuity for snow cover extent data products. Thus, the main objective of our study is to assess the transition of the Terra/Aqua MODIS GWR products into a Suomi National Polar-orbiting Partnership (SNPP, launched in 2011) and JPSS-1 (also known as NOAA-20, launched in 2017) satellites-based standard GWR products. Continuous global reservoir observations are crucial for comprehending long-term reservoir storage and evaporation changes. Furthermore, consistent records serve as the foundation for evaluating the impacts of climate and water management activities on changes in reservoir storage over time.

## Methods

**Fig. 1:**
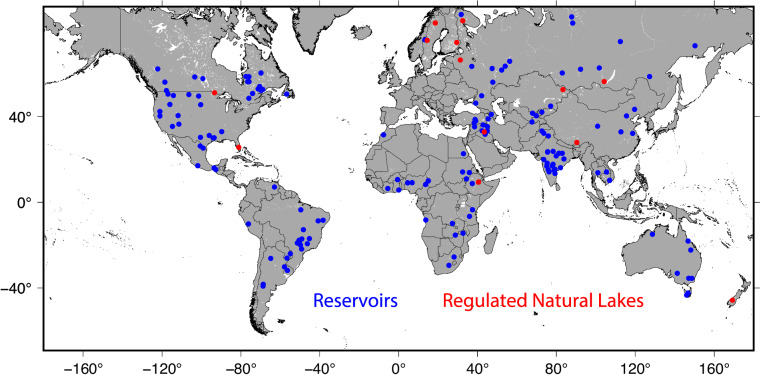
Location of 164 global reservoirs (151 manmade reservoirs (blue) and 13 regulated natural lakes (red)) considered in MODIS/VIIRS water reservoir product suite.

The methods section provides detailed information regarding the input datasets and algorithms utilized in the development of the 8-day (MOD28C2/MYD28C2 and VNP28C2/VJ128C2) and monthly (MOD28C3/MYD28C3 and VNP28C3/VJ128C3) datasets for the MODIS/VIIRS standard GWR products, focusing on 164 global reservoirs (Fig. 1). Throughout the article, the following nomenclature will be used. MOD and MYD refer to the MODIS Terra and Aqua standard GWR products. Similarly, VNP and VJ1 represent the SNPP and JPSS-1 (NOAA-20) standard GWR products. The 8-day reservoir products are denoted with the suffix C2 (e.g., VNP28C2), while the monthly products are indicated with the suffix C3 (e.g., VNP28C3). We have used the terms “NOAA-20” and “JPSS-1” interchangeably in the article; however, they consistently refer to the same satellite throughout the text. For more details about the components of these products, please refer to Table S1 and the following subsections.

### Input data

For the 8-day products (MxD28C2 and Vyy28C2: where x = O for Terra and Y for Aqua, and yy = NP for SNPP and J1 for JPSS1 hereafter), the 8-day surface reflectance data (MxD09Q1^50,51^ and Vyy09H1^52,53^) were obtained to extract reservoir water area. We considered only NIR band due to its high spatial resolution (i.e., 250 m for MODIS and 500 m for VIIRS) and high sensitivity to water. Please note that the difference in MODIS and VIIRS NIR spatial resolution might influence the classification of the edge pixels. The NIR band is widely employed to extract water bodies as water has very low reflectance compared to the surrounding terrestrial bare soil and vegetation^54^. To estimate elevation, we adopted the area-elevation (A-E) relationships from the reliable Global Reservoir Bathymetry Dataset (GRBD^55^), which has undergone rigorous validation against *in-situ* data. Subsequently, the 8-day water area estimations were utilized with the A-E relationships to derive elevation and storage values (further details can be found in Section 2.2). The monthly area was estimated from the composite of the 8-day classifications, which was further used to infer the monthly elevation and storage results via the same A-E relationship. Moreover, we employed the Lake Temperature and Evaporation Model (LTEM^31^) to estimate monthly evaporation rates based on the Penman equation with the heat storage and fetch effects incorporated. To drive the LTEM model, we obtained the 8-day day/night land surface temperature (LST) data (MxD11A2^56,57^ and Vyy21A2^58,59^) from MODIS/VIIRS and meteorological forcing data from the Global Land Data Assimilation System (GLDAS^60^). The input data are summarized in Table S2.

### Algorithm development

This section focuses on utilizing VIIRS products to illustrate the algorithms. The algorithms for the MODIS-based products have been previously described in Li *et al*.^41^. It is worth highlighting that the algorithms for the VIIRS and MODIS GWR products are slightly different. This is because we made refinements to the VIIRS algorithms to reduce the uncertainties associated with ice/snow and terrain shadows (Table S3). It is worth noting that the VIIRS algorithms have been progressively advanced, and these refined algorithms will be applied to the MODIS GWR product in the future. This is expected to further improve the continuity/consistency between MODIS and VIIRS GWR products.

### Algorithm for the 8-Day VIIRS GWR Product

**Fig. 2:**
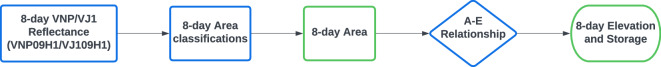
Flowchart for the development of the VIIRS 8-day product (Vyy28C2) components (modified based on Li *et al*.^41^). The green boxes show the output products.

First, the 8-day reservoir area values were extracted from the 8-day 500-m NIR surface reflectance data (i.e., Vyy09H1). During the classification process, an area enhancement algorithm^31^ was implemented to mitigate the underestimation from image contaminations such as clouds and snow/ice. Next, the elevation values for a given reservoir were obtained by applying its A–E relationship^55^ to the area values. Lastly, the reservoir storage values were estimated using Eq. (1).

1 $${V}_{VIIRS}={V}_{c}-\frac{\left({A}_{c}+{A}_{VIIRS}\right)\left({h}_{c}-{h}_{VIIRS}\right)}{2}$$

where *V*_*c*_*, A*_*c*_, and *h*_*c*_ represent storage, area, and water elevation values at capacity and *V*_*VIIRS*_, *A*_*VIIRS*_, and *h*_*VIIRS*_ are the estimated storage, area, and water elevation values, respectively, from VIIRS. It is worth noting that the estimated storage represents the total storage, encompassing both active and dead storage components. Figure 2 shows the overview of the algorithm used for the 8-day product. Table S4 provides detailed information of the reservoir attributes such as location, area, elevation, and storage values at capacity.

Since the extraction of water surface area forms the foundation for estimating elevation and storage, an example (Lake Hawea in New Zealand) highlighting the classification and enhancement algorithms is provided in Figure S1. To ensure full coverage of the water extent, we initially buffered the reservoir shapefile (obtained from HydroLAKES^61^) by 1 km outward. The classification and enhancement operations were performed within this buffered area. For each 8-day period, we selected the Vyy09H1 NIR image that overlapped with the reservoir shapefile (Figure S1a). Subsequently, pixels affected by clouds, cloud shadows, and snow/ice (identified using the Quality Assurance (QA) band of Vyy09H1) were labeled as ‘No Data’. Next, the Otsu thresholding method^62^ was applied to obtain the raw water area classification (Figure S1b). Due to the image contaminations, it is evident that the raw classification underestimates the actual water area. To address this issue, we utilized the enhancement algorithm developed by Zhao *et al*.^31^ to correct the underestimation (Figure S1c). This enhancement algorithm incorporates edge detection techniques and water occurrence images from the Global Surface Water (GSW) dataset^16^ to improve the raw classification. Further details regarding the enhancement algorithm can be found in Zhao *et al*.^31^.

### Improvements of the VIIRS 8-day GWR Algorithm

To improve the data quality assessment for users, we have incorporated contamination percentage values (pertaining to cloud, cloud shadow, and snow/ice) into both the 8-day and monthly area products. For instance, the last field of Vyy28C2 (applicable to Vyy28C3 as well) shows the percentage of contaminated area, which corresponds to the proportion of the reconstructed area from the GSW. These values are obtained by combining the QA information from the reflectance product (e.g., Vyy09H1) with the classification results (i.e., raw water, enhanced water, and not-water). This enables users to comprehensively understand the data quality associated with the products.

The current MODIS product incorporates an enhancement algorithm developed by Zhang *et al*.^23^, whereas the VIIRS product utilizes an enhancement algorithm developed by Zhao *et al*.^31^. While the enhancement algorithm employed in the current MODIS version generally performs well for most reservoirs, it exhibits relatively larger uncertainties when applied to reservoirs located in high-latitude regions^41^. For both enhancement algorithms, one particular challenge is in using a threshold to correct misclassifications. This threshold is estimated using percentile values derived from the edge pixels of a reservoir. Unlike MODIS, the VIIRS enhancement algorithm is based on physical principles and is not dependent on specific parameters. This characteristic enhances its capability to handle edge pixels in high-latitude regions, improving performance^31^.

In order to mitigate area classification errors in reservoirs surrounded by complex and steep terrains, a series of 8-day terrain shadow masks were generated to represent climatological conditions (Figure S2). For example, Figure S2 depicts the climatology of the terrain shadow area within Lake Hawea. The generation of these masks followed the approach developed by Leidman *et al*.^63^, which we further improved by incorporating the average 8-day zenith solar angle during satellite overpass and the Shuttle Radar Topography Mission (SRTM) Digital Elevation Model (DEM). These masks were originally created at a 30-meter resolution and aggregated to match the VNP09H1/VJ109H1 resolution. To implement this algorithm refinement, minor modifications were made to the 8-day image classification code, ensuring that pixels falling within the shadow mask areas were not utilized for raw classification. These modifications were implemented to achieve more accurate raw classifications and improve the representation of water surface areas, addressing aspects that were not adequately covered in the MODIS product.

### Algorithm for Monthly VIIRS GWR Product

**Fig. 3:**
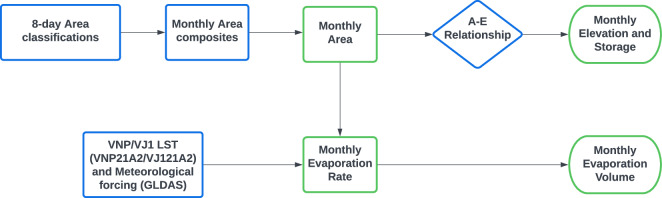
Flowchart for the development of the VIIRS monthly product (Vyy28C3) components (modified based on Li *et al*.^41^). The green boxes show the output products.

The monthly product includes the evaporation rate and volumetric evaporation loss, along with the area, elevation, and storage results. Figure 3 presents the algorithm used to generate the Vyy28C3 monthly product. Firstly, the monthly area values were estimated based on the composite of the 8-day area classifications. Monthly area estimates were generated only when a minimum of two 8-day area estimates were available; otherwise, the data was assigned to a value of “−9999” and categorized as “no-data”. Most of the monthly data were generated based on three to four 8-day estimates in a given month, as illustrated in Figure S3. Secondly, the monthly area was converted into monthly elevation using the A-E relationship. Thirdly, the monthly area, elevation, and reservoir attributes (area, elevation, and storage at capacity, Table S4) were utilized to estimate monthly storage using Eq. 1 (Fig. 3).

The LTEM^31^ was then driven by the meteorological forcing from GLDAS^60^ and constrained by the VIIRS LST data (Vyy21A2) to estimate monthly evaporation rates. Reservoir area and depth values were utilized to account for fetch and heat storage effects in the calculation. Lastly, the monthly evaporative volumetric losses were computed as a function of the evaporation rate and reservoir area. In the case of MxD28C3, the evaporation volume was calculated by multiplying the evaporation rate with the enhanced surface area. However, in high-latitude regions, the enhanced surface area may include both open water and ice-covered areas. As the evaporation loss is negligible for the ice-covered portion, for VIIRS, we improved the estimation of evaporation volume by multiplying evaporation rate with only open water area. The QA information from the reflectance products, along with the classification results, were used to distinguish between open-water pixels and those covered by ice.

## Data Records

The products cover 164 reservoirs, including 151 man-made reservoirs (2,672 km^3^) and 13 regulated natural lakes (23,811 km^3^) (Fig. 1). The total storage capacity of the 151 man-made reservoirs represents 45.82% of the global capacity (in its category as per the Global Reservoir and Dam Database (GranD^1^)). While this number is estimated based on GranD, it may vary when considering different datasets, as each dataset may encompass varying numbers of reservoirs. Table S4 contains the geographical locations and attribute information for the 164 global reservoirs. These reservoirs were selected from GRBD^55^ with areas larger than 25 km^2^. The product is limited to these large reservoirs due to the availability of reliable area-elevation curves at the time when MODIS GWR was first developed. These curves are essential for estimating reservoir storage. Both 8-day and monthly products include reservoir area, elevation, and storage records. However, the monthly product additionally has reservoir evaporation rate and volume records.

The MODIS product (MOD28C2^64^/MYD28C2^65^ & MOD28C3^66^/MYD28C3^67^, Collection 6.1) is publicly released and available at NASA LPDAAC (https://lpdaac.usgs.gov/). More information about the MODIS GWR product is provided at https://modis-land.gsfc.nasa.gov/modgwr.html. Information regarding the data format, extraction code, and outlier removal script is provided in the MODIS GWR user guide (available at https://lpdaac.usgs.gov/documents/1116/MOD28_User_Guide_V61.pdf).

The 8-day (VNP28C2^68^/VJ128C2^69^) and monthly (VNP28C3^70^/VJ128C3^71^) VIIRS Collection 2 GWR product data for the full mission period are also publicly available at NASA LPDAAC. Additional information about the VIIRS GWR product can be found at https://viirsland.gsfc.nasa.gov/Products/NASA/GWR.html. For detailed information on data format, extraction code, and outlier removal scripts, please refer to the VIIRS GWR user guide available at https://lpdaac.usgs.gov/documents/1767/VNP28_UserGuide_V3.pdf. MODIS and VIIRS GWR product time series can be visualized and downloaded at https://landweb.modaps.eosdis.nasa.gov/lake.

## Technical Validations

Since the MODIS product has been validated in Li *et al*.^41^, this article primarily focuses on validating the VIIRS-based products and evaluating the consistencies between VIIRS and MODIS products. For comparing MODIS and SNPP GWR products, an overlapping period from 2012 to 2021 was adopted. For the inter-comparisons among MODIS, SNPP, and JPSS1, the overlapping period from March 2020 to December 2021 was used. The inter-comparison period was short due to the availability of J1 data at the NASA archive during the time of analysis.

Despite the area enhancement process, some unrealistic extreme area values persist occasionally. To eliminate the effects of these outliers, we used three standard deviations as a threshold to remove them and linearly filled the data gaps. However, we only used the linearly interpolated data records to generate line plots/results. This is primarily done to better inspect the consistency visually. It is worth noting that all other results (i.e., scatter and spatial plots) were generated using data records only where both VIIRS and MODIS raw area (outlier removed) observations are available (by not considering the interpolated values). This is because MODIS and VIIRS might have data gaps at different dates; hence, considering interpolated values could lead to uncertainties and biases.

We adopted the coefficient of determination (R^2^), Root Mean Square Error (RMSE), Normalized Root Mean Square Error (NRMSE), and Relative Bias (RB) as metrics to evaluate the consistency of the VIIRS product against the *in-situ* and MODIS observations. The RMSE, NRMSE, and RB were estimated after Eq. 2 to 4, respectively.

2 $$RMSE=\sqrt{\frac{{\sum }_{i=1}^{n}{\left({P}_{i}-{O}_{i}\right)}^{2}}{n}}$$

3 $$NRMSE=\frac{RMSE}{{O}_{max}-{O}_{min}}\times 100 \% $$

4 $$Relative\;Bias\left(RB\right)=\frac{{\sum }_{i=1}^{n}\left({P}_{i}-{O}_{i}\right)}{{\sum }_{i=1}^{n}\left({O}_{i}\right)}\times 100 \% $$

where, *O*_*i*_ is observed/MODIS value, *P*_*i*_ is VIIRS value, and *n* is the total number of observations. *O*_*max*_ and *O*_*min*_ are the maximum and minimum values of observed/MODIS records, respectively.

### Evaluation of VIIRS water surface area

Since the elevation and storage values are estimated by applying A-E relationships to the surface area values, it is crucial to first evaluate the reservoir surface area results. The long-term records of the *in-situ* reservoir area are still lacking on a global scale. Therefore, we compared the monthly VIIRS surface area (VNP28C3/VJ128C3) with MODIS (MOD28C3) and Landsat-based Global Reservoir Surface Area Dataset (GRSAD^18^) during their overlapping periods (2012–2021 with MODIS and 2012–2018 with Landsat). The GRSAD dataset was developed after correcting the water area underestimation of the GSW^16^ dataset caused by both cloud/shadow/ice contamination and the Landsat-7 scan line corrector failure.

**Fig. 4:**
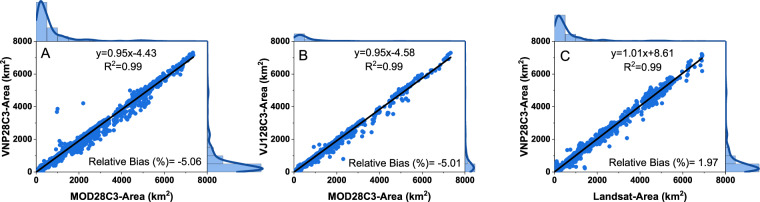
Evaluation of the monthly VIIRS reservoir surface area. (**A**) Comparison of the monthly area estimations between MODIS (Terra) and VIIRS (SNPP) from 2012 to 2021 for the 164 reservoirs; (**B**) Same as (A) but for JPSS-1 (NOAA 20) from March 2020 to December 2021: (**C**) Same as (**A**) but between VIIRS (SNPP) and Landsat from 2012 to 2018. Lake Baikal is excluded from the figure (and from the analysis) due to its extremely large values.

The VIIRS based surface area shows good agreement with the MODIS-based surface area (with an R^2^ value of 0.99). The RB values between the VNP and MOD, as well as between VJ1 and MOD, are around −5% (Fig. 4). The negative bias represents a slight underestimation of the VIIRS area (as compared to the MODIS). This underestimation can be attributed to differences in the sensors (e.g., different spatial resolutions) and algorithms with regard to estimating surface areas. In particular, reservoirs located in regions with steep terrains and small drawdown areas may be affected by the lower resolution of VIIRS (in comparison to MODIS). Moreover, the VIIRS-based product uses a different classification algorithm (from MODIS) and accounts for the terrain shadow effect (which the MODIS-based product does not) (Table S3). The exclusion of the terrain shadow effect in the algorithm could overestimate the surface area in the lakes located in the mountainous region.

There is also a good agreement between VIIRS and Landsat-based surface area, with an R^2^ value of 0.99 and RB value of 1.97% (Fig. 4). Most of the points centered on the regression line (slope = 1.01); however, there are a few disagreements. This could be because the collection dates and methods to derive the monthly area from Landsat and VIIRS differ. It could also be due to the relatively low spatial resolution of VIIRS, which makes it more susceptible to mixed pixels in relatively small reservoirs^41^. Overall, the VIIRS-based area results exhibit satisfactory consistency with other satellite area datasets.

### Evaluation of VIIRS elevation and storage

We evaluated the VIIRS based elevation and storage estimates against MODIS and the *in-situ* observations at 12 Indian reservoirs. The daily and monthly *in-situ* elevation and live storage data were obtained from the Indian Central Water Commission (CWC: http://cwc.gov.in/, accessed on 2^nd^ May 2022) between 2012 and 2021. To validate the 8-day product, we considered the daily *in-situ* observations on the same dates as VIIRS. For the evaluation of the monthly product, we used monthly averaged *in-situ* observations from CWC. We selected the Indian reservoirs for validation purposes due to their large variability, which can better evaluate the efficiency of our algorithm.

Figures S4, S5 show the 8-day elevation and storage validation results. We found good consistency of VIIRS (VNP28C2) elevation with *in-situ* observations, with an average R^2^ value of 0.77, an average RMSE value of 3.34 m, and an average NRMSE value of 13.53% (Figure S4). While elevation performs well at most locations, we noticed cases of overestimations (e.g., Tungabhadra) or underestimations (e.g., Yeleru, Nagarjuna). These biases could be due to misclassifications of the mixed pixels at the reservoir edge and/or errors/uncertainties in the A-E relationships. Moreover, we observed that VIIRS elevation was in good agreement with MODIS (MOD28C2), which can serve as a basis to establish the continuity of MODIS products with VIIRS products. Similar to elevation, the *in-situ* storage variation was satisfactorily captured by VIIRS, with an average R^2^ value of 0.84, an average RMSE value of 0.47 km^3^, and an average NRMSE value of 16.45% (Figure S5). Jayakwadi reservoir showed a maximum R^2^ value of 0.95, and Nagarjuna reservoir showed the least R^2^ value of 0.76. With respect to the continuity perspective, we recognized substantial agreement between MODIS and VIIRS storage estimates.

**Fig. 5:**
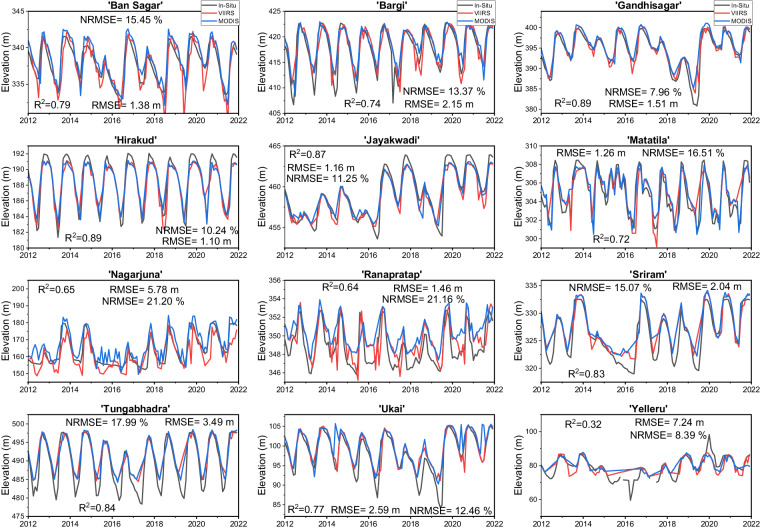
Validation of VIIRS (VNP28C3) monthly elevation values (red) against *in-situ* (black) and MODIS (MOD28C3, blue) observations for twelve Indian reservoirs from 2012 to 2021.

**Fig. 6:**
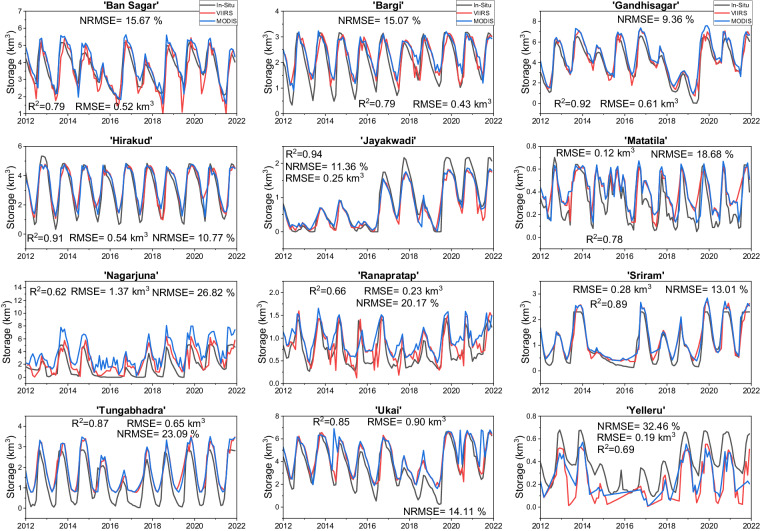
Validation of VIIRS (VNP28C3) monthly storage (red) against *in-situ* (black) and MODIS (MOD28C3, blue) observations for twelve Indian reservoirs from 2012 to 2021.

Similarly, we validated the monthly VIIRS (VNP28C3) elevation and storage products with the *in-situ* and MODIS (MOD28C3) observations. Since the monthly data is generated from the composite of 8-day data, they also manifest robust consistency as 8-day data (Figs. 5, 6). VIIRS monthly reservoir area values were generated from the composited results of three or four 8-day area images from Vyy28C2, reducing the adverse effects of cloud contamination at the 8-day time step and making them smoother. Regarding elevation, the VIIRS results show good agreement against the *in-situ* data with an average R^2^ value of 0.75, an average RMSE value of 2.59 m, and an average NRMSE value of 14.25% (Fig. 5). As for storage, the validation results were also satisfactory, with an average R^2^ value of 0.80, an average RMSE value of 0.50 km^3^, and an average NRMSE value of 17.54% (Fig. 6). While our results demonstrate strong consistency and agreement, we acknowledge the significance of conducting validation at finer units of measurement (such as millions of cubic meters) to improve the precision and practical relevance of our findings in water management applications. Additionally, we have provided NRMSE, a unit-independent metric that provides a transparent assessment of product performance, irrespective of the chosen units of measurement. The consistency between VIIRS and MODIS was exceptional, highlighting that VIIRS-based reservoir products can replace MODIS-based reservoir products after the decommissioning of MODIS.

### Evaluation of VIIRS evaporation rate

We tested the VIIRS (VNP28C3) evaporation rate against the *in-situ* and MODIS (MOD28C3) evaporation rates at Lake Mead and Lake Powell in North America. The validation of the evaporation rate was limited by the availability of high-quality *in-situ* data, which highlights the importance of generating our operational reservoir evaporation data product at a global scale. We obtained the eddy covariance (EC) evaporation rate measurements for Lake Mead between January 2012 and April 2015 from the United States Geological Survey^72^ and for Lake Powell between November 2018 and December 2021 from the Bureau of Reclamation^73^.

**Fig. 7:**
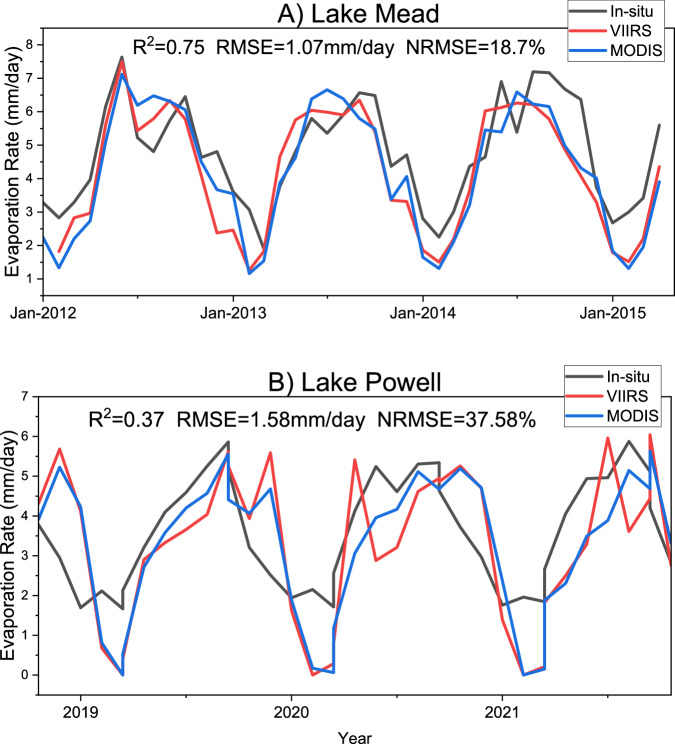
Comparison of the VIIRS (VNP28C3) evaporation rates (red) against *in-situ* data (black) and MODIS (MOD28C3) observations (blue) at (**A**) Lake Mead and (**B**) Lake Powell. *In-situ* data were measured using eddy covariance (EC) measurements for Lake Mead from January 2012 to April 2015 and for Lake Powell from November 2018 to December 2021.

VIIRS captured the seasonality of the evaporation rate at both locations. At Lake Mead, the VIIRS evaporation rate showed good agreement with *in-situ* measurements with R^2^ value of 0.75, RMSE value of 1.07 mm/day, and NRMSE value of 18.7% (Fig. 7). The evaporation rate from VIIRS at Lake Mead also offers nice consistency with MODIS. However, at Lake Powell, we found a low agreement of VIIRS evaporation rate with the *in-situ* data with R^2^ value of 0.37, RMSE value of 1.58 mm/day, and NRMSE value of 37.58% (Fig. 7). These discrepancies in evaporation rates can be attributed to two main factors. First, the *in-situ* eddy covariance data are point measurements, while the VIIRS evaporation rates are for the entire reservoir^31^. Second, the errors associated with the meteorological forcings would affect the Penman equation-based evaporation rates^26^.

Similar to other variables, MODIS and VIIRS evaporation rate estimates show good consistency (Fig. 7). The slight differences in MODIS and VIIRS-based evaporation rate values could be due to differences in sensors and LST products used in LTEM. Zhao *et al*.^31^ validated the MODIS evaporation rate or water temperature profiles at eleven locations located in different climates, which cover a good range of sizes, depths, and elevations and thus are representative for testing the robustness of LTEM. They found a satisfactory performance of MODIS-based evaporation rate against the *in-situ* observations. As most of those *in-situ* data were unavailable after 2012, we could not validate the VIIRS evaporation rate at more locations. However, since MODIS and VIIRS show good consistency for the overlapping period, we anticipate that the VIIRS evaporation rate should be able to offer substantial agreement at other locations.

### Evaluation of VIIRS 8-day elevation against radar altimeter data

We further evaluated the VIIRS 8-day elevation (VNP28C2) against the elevation collected by satellite radar altimeters and MODIS (8-day, MOD28C2). Satellite radar altimeters have been widely used to obtain elevation for global lakes and reservoirs. Therefore, we evaluated our product against the two global operational altimeter products (Hydroweb and G-REALM). We obtained monthly elevation data from 2012 to 2021 from Hydroweb (https://hydroweb.theia-land.fr/, accessed on 5^th^ June 2022^10^) for nine locations in different countries (Volta, Nasser, Kariba, Cahora Bassa, Oahe, Fort Peck, Angostura, Mossul, and Chardarinskoye). The G-REALM data is available at 10-day, 27-day, and 35-day temporal resolutions, depending on the sensor. We used 10-day smoothed elevation data from 2012 to 2021 (https://ipad.fas.usda.gov/cropexplorer/global_reservoir/, accessed on 5^th^ June 2022^9^) for the same nine locations.

**Fig. 8:**
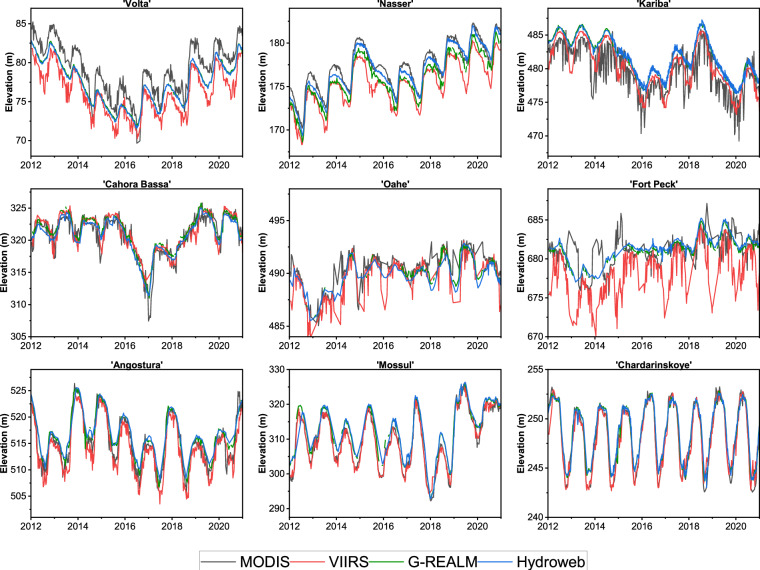
Evaluation of VIIRS (VNP28C2) 8-day elevation (red) against radar altimetry products (Hydroweb (blue) and G-REALM (green)) and the MODIS based (MOD28C2) product (black) from 2012 to 2021 at nine different locations across the world.

Overall, the VIIRS product and radar altimetry products agree well (Fig. 8). For instance, the Angostura reservoir in Mexico showed good consistency among different products. Similarly, the results were consistent and satisfactory for the Mossul reservoir in Iraq and the Chardarinskoye reservoir, located in Kazakhstan. However, we observed some bias and inconsistency for Fort Peck and Oahe reservoirs in the USA. This is mainly due to the bias in the 8-day area classification, which is used to estimate elevation using the A-E relationship. Gao *et al*.^22^ addressed a similar issue caused by mixed pixels along the reservoir shores. Since Fort Peck Reservoir is a sinuous water body in shape, many of the MODIS/VIIRS grid cells over the reservoir are border cells partially covered by water and partially covered by land. This can lead to inconsistencies in area estimation and result in biased elevation.

In general, the MODIS/VIIRS product suite can offer additional benefits over traditional radar altimetry products by improving temporal resolution (at an 8-day) and providing continuous long-term data^41^. While a majority of the locations exhibit good agreement between VIIRS and MODIS elevation, at some places, MODIS shows larger elevation values than VIIRS (e.g., Volta, Nasser). This can be due to differences in sensors, raw classification, and enhancement algorithms used in both products. On the other hand, the inconsistency in raw data (without linear interpolation) seems minimal (Figure S6). Overall, we found good consistency of VIIRS with radar altimeter and MODIS data.

### Consistency between VIIRS (VNP) and MODIS Terra (MOD) GWR products

To determine the continuity between VIIRS (VNP) and MODIS Terra product (MOD) at each of the 164 reservoirs, we calculated the R^2^, NRMSE (%), and RB (%) between VIIRS and MODIS-based monthly storage, evaporation rate, and evaporation volume estimates. The R^2^ for monthly storage was found to be high (0.5–0.99) at most locations (Figure S7). For instance, R^2^ was more than 0.5 for 100 out of the 164 reservoirs. However, in some high-latitude reservoirs, the R^2^ values are relatively low, indicating the differences in storage due to the influence of terrain shadow. The evaporation rate shows greater consistency between VIIRS and MODIS, with high R^2^ at almost all locations. Similarly, the evaporation volume also exhibits R^2^ > 0.5 at most locations. In addition, we estimated the R^2^ between VIIRS and MODIS-based 8-day area, elevation, and storage estimates (Figure S8). Like monthly results, we found relatively high R^2^ in tropical and south regions but low R^2^ in high-latitude regions (Figure S8).

The RB for monthly storage in most mid-latitude reservoirs ranged from −10% to 0 (according to Figure S9). These negatives indicate that the VIIRS storage values are underestimated compared to MODIS, mainly due to the implementation of the new algorithms. Furthermore, reservoirs in high latitude or mountainous regions exhibited a higher degree of underestimation (more negative RB) in storage, attributed to the factors described earlier (e.g., terrain shadow). Regarding the evaporation rate, the RB typically ranged from −15% to 15% at most locations. The RB for the evaporation rate exhibits divergent spatial patterns, displaying a negative RB in tropical regions and a positive bias in high-latitude areas. This discrepancy can be attributed to the utilization of different LST data for estimating the evaporation rate in MODIS (MOD11A2) and VIIRS (VNP21A2). The RB for evaporation volume exhibits a similar pattern as the evaporation rate, with slight differences in high-latitude regions. The bias in MODIS and VIIRS evaporation volume can be linked to differences in the area considered to estimate evaporation volume. While MODIS considers the total water area while calculating the evaporation volume, the VIIRS only considers the open water area. Moreover, the RB for the 8-day area, elevation, and storage also indicates the underestimation of VIIRS-based estimates compared to MODIS (Figure S10).

**Fig. 9:**
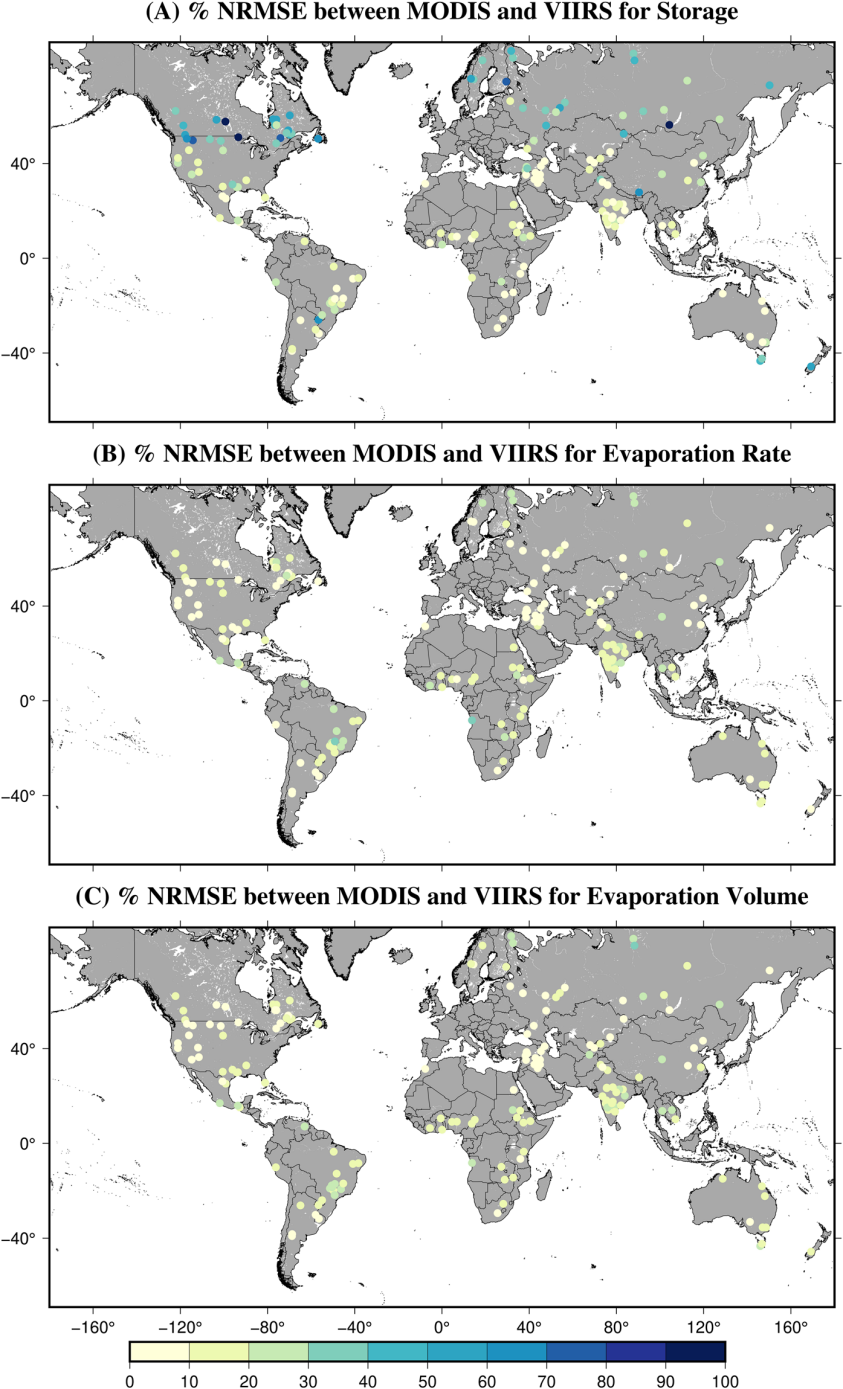
% NRMSE values between VIIRS (VNP28C3) and the MODIS Terra product (MOD28C3) for (**A**) Monthly Storage, (**B**) Evaporation Rate, and (**C**) Evaporation Volume from 2012 to 2021 at 164 global reservoirs.

We further investigated the continuity through the lens of the NRMSE (Fig. 9). Regarding monthly storage, the NRMSE values were relatively low, ranging from around 10% to 20%, with a few exceptions in high-latitude reservoirs. Additionally, a couple of reservoirs in Australia and New Zealand exhibited higher NRMSE values along with negative RB values, indicating an underestimation of VIIRS storage. In contrast, the NRMSE values for evaporation rate and evaporation volume were consistently low across most reservoirs worldwide. The results for NRMSE for 8-day area, elevation, and storage depict a similar pattern as monthly storage, highlighting the underestimation of VIIRS in high-latitude regions (Figure S11). In conclusion, the VIIRS and MODIS Terra products demonstrated good consistency overall.

### Consistency between VIIRS and MODIS Aqua (MYD) GWR products

**Fig. 10:**
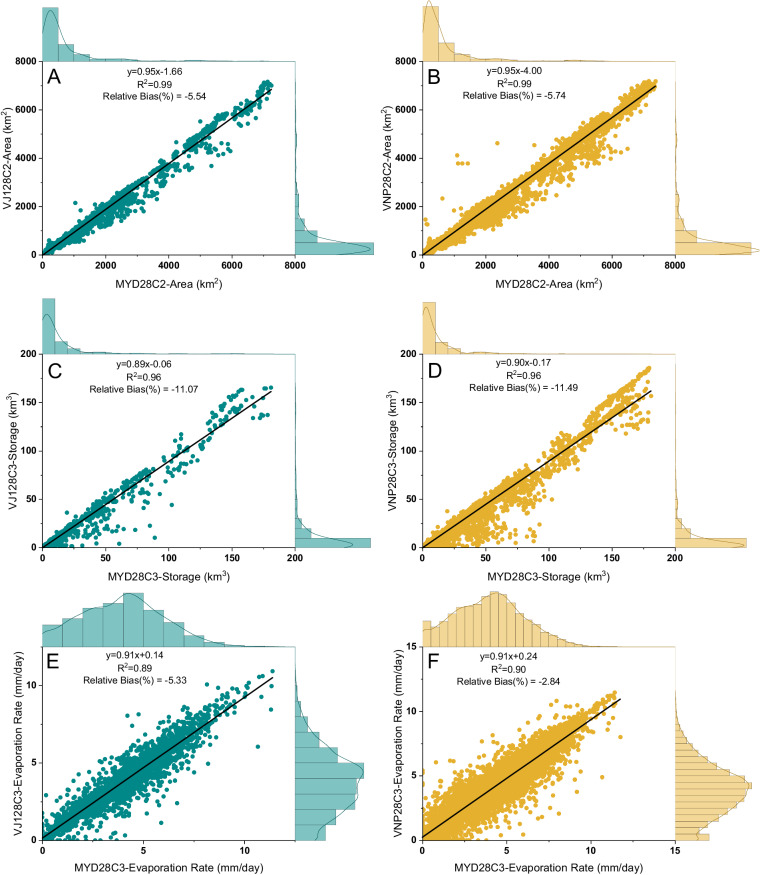
Comparison of the 8-day surface area, monthly reservoir storage, and monthly evaporation rate between MODIS Aqua (MYD) and VIIRS (JPSS-1/VJ1) from March 2020 to December 2021 (left column), as well as between MODIS Aqua and VIIRS (SNPP/VNP) from 2012 to 2021 (right column). Lake Baikal is excluded from the figure (and from the analysis) due to its extremely large values.

The majority of previous results focused on the comparison between SNPP (VNP) and Terra MODIS (MOD). Considering MODIS Terra (morning) and Aqua (afternoon) have different overpass times, we further evaluated consistency among VIIRS (both SNPP and JPSS-1/NOAA 20) and Aqua MODIS (MYD). Figure 10 illustrates the results for the 8-day area, monthly storage, and monthly evaporation rate for both SNPP (VNP) and JPSS-1 (VJ1) products compared to the MODIS Aqua product (MYD).

We observed a strong agreement between the 8-day VIIRS area from SNPP and JPSS-1 with that of MYD (Fig. 10A,B), yielding high R^2^ values of 0.99. The RB values for VJ1 and VNP were −5.54% and −5.74%, respectively. Likewise, the monthly storage estimates (derived from the monthly area composites) exhibited a high level of consistency, with RBs of −11.07% and −11.49% and R^2^ values of 0.96 for both VJ1 and VNP (Fig. 10C,D). Furthermore, the R^2^ values between the MODIS and VIIRS-based monthly evaporation rates were 0.89 for VJ1 and 0.90 for VNP (Fig. 10E,F). The RB for VJ1 was slightly higher (−5.53%) than VNP (−2.84%).

In general, our findings reveal a strong concurrence between the MODIS products (MOD and MYD) and VIIRS products (VNP and VJ1). This underscores the potential for seamless continuity, regardless of the varying overpassing times. While MODIS is nearing the end of its operational life, VIIRS can be effectively used to generate long-term reservoir records. By combining VIIRS records with MODIS data, a long-term (20 + years), consistent, continuous, and coherent global water reservoir dataset can be created.

### Consistency between SNPP (VNP) and JPSS-1 (VJ1) GWR products

NASA plans to provide MODIS Land continuity products through the VIIRS instruments, specifically SNPP and NOAA 20 (previously known as JPSS-1). The latest satellite in the JPSS series, JPSS-2/NOAA-21, was launched in November 2022. Furthermore, the launching of JPSS-3 and JPSS-4 has been planned for 2027 and 2032, respectively. Thus, ensuring continuity among the VIIRS products is essential for seamless data integration. In this section, we compared VNP (SNPP) and VJ1 (JPSS-1) based 8-day surface area and monthly evaporation loss from March 2020 to December 2021.

**Fig. 11:**
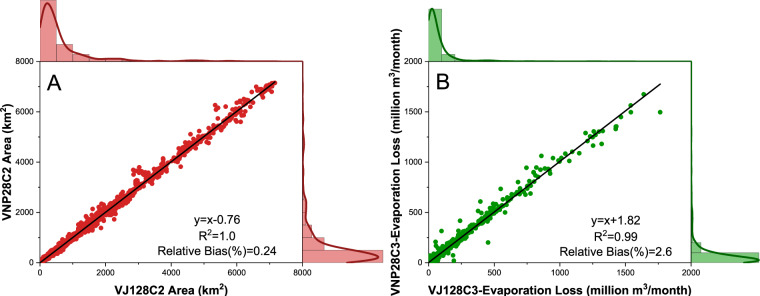
The comparison of the (**A**) 8-day surface area and (**B**) monthly evaporation loss between VIIRS SNPP (VNP) and JPSS-1/NOAA 20 (VJ1) from March 2020 to December 2021. Lake Baikal is excluded from the figure (and from the analysis) due to its extremely large values.

Since VNP and VJ1 products utilize the same type of VIIRS sensors and algorithms, there is a substantially larger agreement between the two as compared to MODIS versus VIIRS. The R^2^ values for surface area and evaporation loss were 1.00 and 0.99, respectively, highlighting a very strong correlation (Fig. 11). The RB values for surface area and evaporation loss were under 3%, indicating a seamless continuity between SNPP and JPSS reservoir products. In conclusion, JPSS (VJ1) serves as an effective successor to SNPP (VNP) for creating long-term records in the future. The VIIRS products align well with each other and can be combined with MODIS data to provide valuable insights into reservoir dynamics on a global scale.

### Product continuity for long-term trend analysis

Understanding long-term trends in reservoir storage is crucial for water resource managers as it enables them to assess water availability over time and make well-informed decisions regarding water allocation, conservation, and future planning. Given the importance of long-term data and trends in reservoir storage, our goal is to evaluate the feasibility of integrating VIIRS with MODIS to ensure the continuous analysis of long-term trends even after the decommissioning of MODIS. By achieving this integration, we aim to maintain a seamless and consistent assessment of reservoir trends, which is essential for effective water resource management.

To do so, we first removed the outliers to avoid biased trends from unrealistic extreme values. We then filled the data gaps via linear interpolation to ensure continuous data records. Since MODIS and VIIRS GWR product variables have differences in absolute values due to differences in sensors and algorithms (see sections 2.2 and 4.1–4.6), we used their anomalies to estimate trends. Specifically, for VIIRS, we calculated the anomalies by subtracting the monthly mean values from 2012 to 2021 from the corresponding VIIRS storage values for each month. Since MODIS contains longer data records than VIIRS, to keep it consistent with VIIRS, we constructed the MODIS storage anomalies by removing MODIS seasonality derived from the same period (i.e., 2012–2021) from MODIS storage values during 2000–2021. This approach ensured that both VIIRS and MODIS anomalies were estimated from the same baseline, allowing for unbiased comparisons between the two datasets.

To assess the long-term trend, we employed a nonparametric Mann-Kendall trend test^74,75^ and Sen’s slope method^76^ at a 5% significance level. First, we estimated the trend (Sen’s slope) in monthly MODIS storage anomalies from 2000 to 2021 (hereafter MOD). Subsequently, we merged the MODIS storage anomalies from 2000 to 2012 with VIIRS (VNP28C3) storage anomalies from 2012 to 2021 (hereafter MOD + VNP). Using the same method as in MOD, we then estimated the Sen’s slope in MOD + VNP to determine whether the long-term trend remained sustained when the MODIS anomalies were replaced with VIIRS anomalies during the overlapping period (2012–2021).

**Fig. 12:**
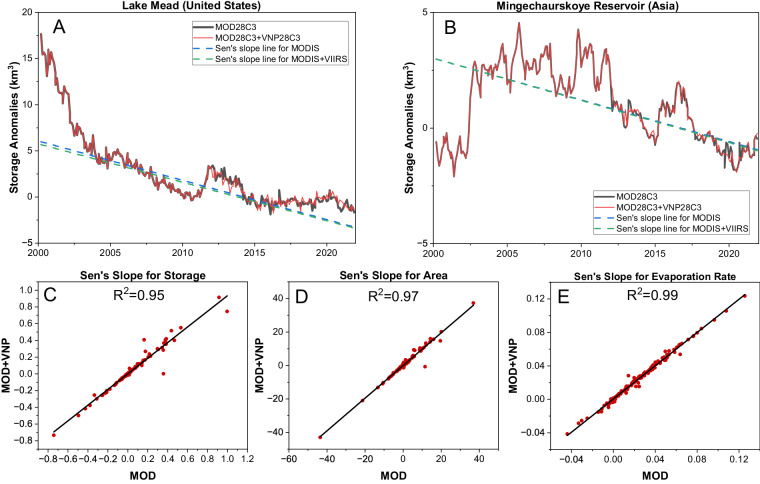
Trend analysis of storage anomalies using Mann‐Kendall and Sen’s slope method for the period of 2012–2021 at (**A**) Lake Mead (North America) and (**B**) Mingechaurskoye reservoir (Asia). The black line shows the monthly storage anomalies from MOD28C3. The red line shows the combined monthly storage anomalies from MODIS (from 2000–2012) and VIIRS (from 2012–2021). The blue dotted line shows Sen’s slope for MODIS, and the green dotted line shows Sen’s slope for the MODIS + VIIRS combined storage anomalies. (**C**–**E**) Comparison of the Sen’s slope values between MODIS (MOD) and MODIS + VIIRS (MOD + VNP) for all 164 reservoirs with respect to (**C**) storage anomalies, (**D**) area anomalies, and (**E**) evaporation rate anomalies.

Figure 12A,B presents long-term trends (Sen’s slope) in storage anomalies for MOD (MOD28C3) and MOD + VNP (MOD28C3 + VNP28C3) for two selected locations: Lake Mead in North America and Mingechaurskoye reservoir in Asia. In both cases, the Sen’s slope lines for MOD and MOD + VNP are nearly identical, indicating that the long-term trends are sustained even when replacing MODIS observations with VIIRS. To ensure that these results are not case-specific and are valid for other locations and other variables (i.e., area, evaporation rate), we conducted a comprehensive trend analysis on monthly storage, area, and evaporation rate anomalies for 164 reservoirs (Fig. 12C–E). The analysis revealed a strong agreement between the Sen’s slope of MOD and MOD + VNP, as evidenced by high R^2^ values (0.95 for storage, 0.97 for surface area, and 0.99 for evaporation rate). These findings underscore the suitability of VIIRS as a viable successor to MODIS, effectively capturing reservoir dynamics with satisfactory accuracy and continuity.

### Limitations and sources of uncertainties

The limited spatial resolutions of MODIS and VIIRS necessitated the inclusion of predominantly larger reservoirs in the GWR standard products. However, it is more appropriate for smaller reservoirs to utilize missions that offer higher spatial resolutions, such as Landsat and Sentinel-2. The uncertainty associated with surface area estimation primarily arises from the raw classification and enhancement algorithms. The presence of mixed pixels at the borders of reservoirs can introduce uncertainties in the raw classification results^31^, especially for lakes characterized by a significant shoreline-to-area ratio^22^. In addition, reservoirs located in regions with steep terrain and small drawdown areas may be affected by the misclassification of the edge pixels. Another limitation of VIIRS/MODIS arises in areas with persistent cloud cover (such as the Western Ghats in India) or regions with pronounced monsoon seasonality. In such cases, the VIIRS/MODIS surface area may not be available for an extended period.

The quality of the water occurrence image also impacts the enhancement algorithm. For instance, in high-latitude regions, the water occurrence image exhibits minimal variation in surface area dynamics, indicated by a left-skewed distribution of occurrence values. Consequently, pixels with low occurrence values exhibit relatively high levels of uncertainty^41^. In addition, lake ice cover also influences the classification by generating incorrect threshold values, leading to overestimation of surface areas in high latitude regions.

The uncertainties related to reservoir surface area, as discussed earlier, can propagate into uncertainties in elevation and storage estimates. In particular, storage uncertainties can arise from factors such as uncertainties in the A-E relationship and reservoir configuration. To estimate storage (i.e., Eq. 1), the area, elevation, and storage values at full capacity are required. However, variations in these input parameters can be found across different sources, and they may also change dynamically due to sedimentation. The uncertainties in the A-E relationship coefficients can impact elevation estimates. For example, assuming a linear A-E relationship may introduce bias, particularly when a non-linear relationship better fits in certain cases^55^.

The sources of uncertainty in evaporation rate estimation primarily encompass uncertainty in GLDAS forcing data and uncertainty in model structure and parameters^30,31^. Zhao and Gao^30^ found that the evaporation rate values differ when the LTEM was driven by three different forcing datasets (TerraClimate, GLDAS, and North American Land Data Assimilation System (NLDAS)). These discrepancies were attributed to the incoming shortwave radiation and other meteorological variables. The forcing data utilized in this study (i.e., GLADS-2.1) consists of land-based meteorological records. However, we have not accounted for differences in wind speeds between the reservoir and land regions, which may introduce some uncertainties^77^. Alternatively, uncertainty in the model structure and parameters may arise from simplified assumptions. For instance, the formulation of the light attenuation coefficient (λPAR) in LTEM is simplified as a constant^31^. However, λPAR is sensitive to many time-varying factors that are difficult to measure, such as suspended solids, phytoplankton concentration levels, and the spectral distribution of solar radiation^78,79^.

It is important to acknowledge two limitations of LTEM, which is the core algorithm for evaporation rate. Firstly, the current version of LTEM does not incorporate convective heat flux. In cases with a significant temperature difference between inflow and outflow in reservoirs, the convective energy flux can be substantial and may affect the accuracy of evaporation rate estimations^31^. Secondly, when there is ice over the reservoir, Zhao *et al*.^31^ assumed the absence of heat fluxes between the air and water, as well as the absence of shortwave radiation infiltration. Despite these uncertainties, our product demonstrates good agreement with *in-situ* data and contributes to developing a global reservoir monitoring product, which is crucial for creating effective water governance.

### Future directions

Optical sensors like MODIS and VIIRS provide reasonable water area estimates, but their effectiveness can be hindered by cloud cover and uncertainties near complex reservoir shorelines. In contrast, synthetic aperture radar (SAR) can penetrate clouds, making it a valuable tool for reservoir monitoring under various weather conditions^80,81^. Therefore, a multi-sensor approach, combining optical and SAR data, offers a more robust solution for comprehensive reservoir monitoring throughout the year. Recent efforts have focused on integrating near-nadir active SAR data with optical and altimeter data from platforms such as Jason and the Ice, Cloud, and Land Elevation Satellite (ICESat) to infer surface water dynamics^80,82–84^. Additionally, ongoing investigations are exploring the potential of the Surface Water and Ocean Topography (SWOT) mission to enhance the development of multi-sensor reservoir products^85–88^. In the future, the development of multi-satellite reservoir products—with improved spatial and temporal resolutions—will be crucial for monitoring reservoirs of all sizes in all weather conditions.

### Supplementary information

Supplementary Information

## Data Availability

Codes used for extracting the 8-day and monthly VIIRS data, along with the outlier removal and gap-filling scripts, are available through the GitHub repository: https://github.com/beingdeepshah/MODIS_VIIRS_GWR_Codes.

## References

[1] Lehner B (2011). High-resolution mapping of the world’s reservoirs and dams for sustainable river-flow management. Front Ecol Environ.

[2] Mulligan M, van Soesbergen A, Sáenz L (2020). GOODD, a global dataset of more than 38,000 georeferenced dams. Scientific Data.

[3] Wang J (2022). GeoDAR: georeferenced global dams and reservoirs dataset for bridging attributes and geolocations. Earth Syst Sci Data.

[4] Zhang, A. T. & Gu, V. X. Global Dam Tracker: A database of more than 35,000 dams with location, catchment, and attribute information. *Sci Data* **10**, (2023). 10.1038/s41597-023-02008-2 PMC9950439 36823207

[5] Song C (2022). A comprehensive geospatial database of nearly 100 000 reservoirs in China. Earth Syst Sci Data.

[6] Wolf AT (1999). International River Basins of the World. Int J Water Resour Dev.

[7] Birkett CM (1994). Radar altimetry: A new concept in monitoring lake level changes. Eos, Transactions American Geophysical Union.

[8] Birkett, C. M. The contribution of TOPEX/POSEIDON to the global monitoring of climatically sensitive lakes. *J Geophys Res* **100**, (1995).

[9] Birkett, C., Reynolds, C., Beckley, B. & Doorn, B. From research to operations: The USDA global reservoir and lake monitor. *Coastal Altimetry* 19–50, 10.1007/978-3-642-12796-0_2 (2011).

[10] Crétaux JF (2011). SOLS: A lake database to monitor in the Near Real Time water level and storage variations from remote sensing data. Advances in Space Research.

[11] Schwatke C, Dettmering D, Bosch W, Seitz F (2015). DAHITI - An innovative approach for estimating water level time series over inland waters using multi-mission satellite altimetry. Hydrol Earth Syst Sci.

[12] Donchyts G (2016). Earth’s surface water change over the past 30 years. Nat Clim Chang.

[13] Khandelwal A (2017). An approach for global monitoring of surface water extent variations in reservoirs using MODIS data. Remote Sens Environ.

[14] Khandelwal, A. *et al*. ReaLSAT, a global dataset of reservoir and lake surface area variations. *Sci Data* **9**, (2022).

[15] Ling F (2020). Monitoring surface water area variations of reservoirs using daily MODIS images by exploring sub-pixel information. ISPRS Journal of Photogrammetry and Remote Sensing.

[16] Pekel JF, Cottam A, Gorelick N, Belward AS (2016). High-resolution mapping of global surface water and its long-term changes. Nature.

[17] Yao, F., Wang, J., Wang, C. & Crétaux, J. F. Constructing long-term high-frequency time series of global lake and reservoir areas using Landsat imagery. *Remote Sens Environ* **232**, (2019).

[18] Zhao G, Gao H (2018). Automatic Correction of Contaminated Images for Assessment of Reservoir Surface Area Dynamics. Geophys Res Lett.

[19] Li Y, Zhao G, Allen GH, Gao H (2023). Diminishing storage returns of reservoir construction. Nature Communications.

[20] Yao F (2023). Satellites reveal widespread decline in global lake water storage. Science (1979).

[21] Busker T (2019). A global lake and reservoir volume analysis using a surface water dataset and satellite altimetry. Hydrol Earth Syst Sci.

[22] Gao, H., Birkett, C. & Lettenmaier, D. P. Global monitoring of large reservoir storage from satellite remote sensing. *Water Resour Res* **48** (2012).

[23] Zhang S, Gao H, Naz BS (2014). Monitoring reservoir storage in South Asia from multisatellite remote sensing. Water Resour Res.

[24] Zhang, S. & Gao, H. Using the digital elevation model (DEM) to improve the spatial coverage of the MODIS based reservoir monitoring network in South Asia. *Remote Sens (Basel)* **12**, (2020).

[25] Zhao G, Li Y, Zhou L, Gao H (2022). Evaporative water loss of 1.42 million global lakes. Nat. Commun..

[26] Zhao, B. *et al*. Evaluating Enhanced Reservoir Evaporation Losses From CMIP6-Based Future Projections in the Contiguous United States. *Earths Future* **11**, (2023).

[27] Friedrich K (2018). Reservoir evaporation in the Western United States: Current science, challenges, and future needs. Bull. Am. Meteorol. Soc..

[28] Wang W (2018). Global lake evaporation accelerated by changes in surface energy allocation in a warmer climate. Nat Geosci.

[29] Zhang H, Gorelick SM, Zimba PV, Zhang X (2017). A remote sensing method for estimating regional reservoir area and evaporative loss. J Hydrol (Amst).

[30] Zhao G, Gao H (2019). Estimating reservoir evaporation losses for the United States: Fusing remote sensing and modeling approaches. Remote Sens Environ.

[31] Zhao, G., Gao, H. & Cai, X. Estimating lake temperature profile and evaporation losses by leveraging MODIS LST data. *Remote Sens Environ* **251**, (2020).

[32] Tian, W. *et al*. Estimation of global reservoir evaporation losses. *J. Hydrol*. **607** (2022).

[33] Lowe LD, Webb JA, Nathan RJ, Etchells T, Malano HM (2009). Evaporation from water supply reservoirs: An assessment of uncertainty. J. Hydrol..

[34] Dias NL, Hoeltgebaum LEB, Santos I (2023). STAEBLE: A Surface-Temperature- and Available-Energy-Based Lake Evaporation Model. Water Resour Res.

[35] Fisher JB (2023). Remotely sensed terrestrial open water evaporation. Scientific Reports.

[36] Althoff, D., Rodrigues, L. N. & da Silva, D. D. Evaluating evaporation methods for estimating small reservoir water surface evaporation in the Brazilian savannah. *Water (Switzerland)* **11** (2019).

[37] Meng X, Liu H, Du Q, Xu L, Liu Y (2020). Evaluation of the performance of different methods for estimating evaporation over a highland open freshwater lake in mountainous area. Water (Switzerland).

[38] Mhawej M, Fadel A, Faour G (2020). Evaporation rates in a vital lake: a 34-year assessment for the Karaoun Lake. Int J Remote Sens.

[39] Friedrich, K., Grossman, R., … J. H.-B. of the & 2018, undefined. Reservoir evaporation in the Western United States: current science, challenges, and future needs. *American Meteorological Society*10.1175/BAMS-D-15-00224.1 (2018).

[40] Shah D, Zhao G, Li Y, Singh VP, Gao H (2024). Assessing Global Reservoir-Based Hydrological Droughts by Fusing Storage and Evaporation. Geophys Res Lett.

[41] Li Y (2021). NASA’s MODIS/VIIRS Global Water Reservoir Product Suite from Moderate Resolution Remote Sensing Data. Remote Sensing.

[42] Román MO (2024). Continuity between NASA MODIS Collection 6.1 and VIIRS Collection 2 land products. Remote Sens Environ.

[43] Doelling, D., Morstad, D., … R. B.-W. M. & 2011, undefined. Algorithm theoretical basis document (ATBD) for deep convective cloud (DCC) technique of calibrating GEO sensors with Aqua-MODIS for GSICS. *gsics.atmos.umd.eduDR Doelling, D Morstad, R Bhatt, B ScarinoWorld Meteorological Organization, Geneva, 2011•gsics.atmos.umd.edu* (2011).

[44] Vermote E, Kaufman YJ (1995). Absolute calibration of AVHRR visible and near-infrared channels using ocean and cloud views. Int J Remote Sens.

[45] Frey RA, Ackerman SA, Holz RE, Dutcher S, Griffith Z (2020). The continuity MODIS-VIIRS cloud mask. Remote Sensing.

[46] Platnick S (2020). The NASA MODIS-VIIRS Continuity Cloud Optical Properties Products. Remote Sensing.

[47] Riggs G, Hall D (2020). Continuity of MODIS and VIIRS snow cover extent data products for development of an earth science data record. Remote Sensing.

[48] Skakun S, Justice CO, Vermote E, Roger JC (2018). Transitioning from MODIS to VIIRS: an analysis of inter-consistency of NDVI data sets for agricultural monitoring. International journal of remote sensing,.

[49] Liu Y (2017). Evaluation of the VIIRS BRDF, Albedo and NBAR products suite and an assessment of continuity with the long term MODIS record. Remote Sens Environ.

[50] Vermote, E. MYD09Q1 MODIS/Aqua Surface Reflectance 8-Day L3 Global 250m SIN Grid V006 [Data set]. *NASA EOSDIS Land Processes DAAC* (2015).

[51] Vermote, E. MOD09Q1 MODIS/Terra Surface Reflectance 8-Day L3 Global 250m SIN Grid V006 [Data set]. *NASA EOSDIS Land Processes DAAC* (2015).

[52] Vermote E, Franch B, Claverie M (2016). VIIRS/NPP Surface Reflectance 8-Day L3 Global 500m SIN Grid V001 [Data set]. NASA EOSDIS Land Processes DAAC. Accessed.

[53] Vermote E, Franch B, Claverie M (2023). NASA EOSDIS Land Processes Distributed Active Archive Center.

[54] sensing, S. M.-I. journal of remote & 1996, undefined. The use of the Normalized Difference Water Index (NDWI) in the delineation of open water features. *Taylor & Francis* **17**, 1425–1432 (1996).

[55] Li, Y., Gao, H., Zhao, G. & Tseng, K. H. A high-resolution bathymetry dataset for global reservoirs using multi-source satellite imagery and altimetry. *Remote Sens Environ* **244**, (2020).

[56] Wan Z., H. S., H. G. MYD11A2 MODIS/Aqua Land Surface Temperature/Emissivity 8-Day L3 Global 1km SIN Grid V006 [Data set]. *NASA EOSDIS Land Processes DAAC* (2015).

[57] Wan Z., H. S., H. G. MOD11A2 MODIS/Terra Land Surface Temperature/Emissivity 8-Day L3 Global 1km SIN Grid V006 [Data set]. *NASA EOSDIS Land Processes DAAC* (2015).

[58] Hulley, G. & Hook, S. VIIRS/NPP Land Surface Temperature and Emissivity 8-Day L3 Global 1km SIN Grid V001 [Data set]. *NASA EOSDIS Land Processes DAAC*. (2018).

[59] Hulley G, Hook S (2023). NASA EOSDIS Land Processes Distributed Active Archive Center.

[60] Rodell M (2004). The Global Land Data Assimilation System. Bull Am Meteorol Soc.

[61] Messager, M. L., Lehner, B., Grill, G., Nedeva, I. & Schmitt, O. Estimating the volume and age of water stored in global lakes using a geo-statistical approach. *Nat Commun* **7** (2016). 10.1038/ncomms13603 PMC5171767 27976671

[62] Otsu N (1979). A threshold selection method from gray level histograms. IEEE Trans Syst Man Cybern.

[63] Leidman SZ, Rennermalm ÅK, Lathrop RG, Cooper MG (2021). Terrain-Based Shadow Correction Method for Assessing Supraglacial Features on the Greenland Ice Sheet. Frontiers in Remote Sensing.

[64] Gao H (2021). NASA EOSDIS Land Processes Distributed Active Archive Center.

[65] Gao H (2021). NASA EOSDIS Land Processes Distributed Active Archive Center.

[66] Gao H (2021). NASA EOSDIS Land Processes Distributed Active Archive Center.

[67] Gao H (2021). NASA EOSDIS Land Processes Distributed Active Archive Center.

[68] Gao H (2024). NASA EOSDIS Land Processes Distributed Active Archive Center.

[69] Gao H (2024). NASA EOSDIS Land Processes Distributed Active Archive Center.

[70] Gao H (2024). NASA EOSDIS Land Processes Distributed Active Archive Center.

[71] Gao H (2024). NASA EOSDIS Land Processes Distributed Active Archive Center.

[72] Moreo MT (2015). Evaporation data from Lake Mead and Lake Mohave, Nevada and Arizona, March 2010 through April 2015. S Geological Survey, org/.

[73] Holman, K., P. C., J. R., H. J. L., V. J. Evaporation from Lake Powell. *In-situ* Monitoring between 2018 and 2021 [Dataset]. *Bureau of Reclamation, Denver, Colorado, USA* (2022).

[74] Mann HB (1945). Nonparametric tests against trend. Econometrica.

[75] Kendall MG (1975). Rank correlation methods. 4th edition, Charles Griffin, London. References Scientific Research Publishing.

[76] Sen PK (1968). Estimates of the regression coefficient based on Kendall’s tau. J Am Stat Assoc.

[77] Schwab DJ, Morton JA (1984). Estimation of Overlake Wind Speed from Overland Wind Speed: A Comparison of Three Methods. J Great Lakes Res.

[78] Lee Z-P (2005). Diffuse attenuation coefficient of downwelling irradiance: An evaluation of remote sensing methods. Wiley Online Library.

[79] Pinhassi J, DeLong EF, Béjà O, González JM, Pedrós-Alió C (2016). Marine Bacterial and Archaeal Ion-Pumping Rhodopsins: Genetic Diversity, Physiology, and Ecology. Microbiology and Molecular Biology Reviews.

[80] Das P (2022). Reservoir Assessment Tool 2.0: Stakeholder driven improvements to satellite remote sensing based reservoir monitoring. Environmental Modelling & Software.

[81] Biswas, N., Hossain, F., Bonnema, M., Software, H. L.-… M. & & 2021, undefined. Towards a global Reservoir Assessment Tool for predicting hydrologic impacts and operating patterns of existing and planned reservoirs. *Elsevier*.

[82] Perin V, Tulbure MG, Gaines MD, Reba ML, Yaeger MA (2022). A multi-sensor satellite imagery approach to monitor on-farm reservoirs. Remote Sens Environ.

[83] Pham-Duc B, Prigent C, Aires F (2017). Surface Water Monitoring within Cambodia and the Vietnamese Mekong Delta over a Year, with Sentinel-1 SAR Observations. Water.

[84] Chen T (2022). Monitoring global reservoirs using ICESat-2: Assessment on spatial coverage and application potential. J Hydrol (Amst.

[85] Bonnema M, Hossain F (2019). Assessing the Potential of the Surface Water and Ocean Topography Mission for Reservoir Monitoring in the Mekong River Basin. Water Resour Res.

[86] Munier S, Polebistki A, Brown C, Belaud G, Lettenmaier DP (2015). SWOT data assimilation for operational reservoir management on the upper Niger River Basin. Water Resour Res.

[87] Xiong J (2023). On the capabilities of the SWOT satellite to monitor the lake level change over the Third Pole. Environmental Research Letters.

[88] Biancamaria, S., Lettenmaier, D. P. & Pavelsky, T. M. The SWOT Mission and Its Capabilities for Land Hydrology. 117–147, 10.1007/978-3-319-32449-4_6 (2016).

