# Supplementary material for: Transitioning from MODIS to VIIRS Global Water Reservoir Product
Source: Sci Data. 2024 Feb 15;11:209. doi: 10.1038/s41597-024-03028-2 (PMC10869837; doi:10.1038/s41597-024-03028-2)
Supplement: Supplementary file 1 — Supplementary Information [file 41597_2024_3028_MOESM1_ESM.docx]

**Supplemental Material**

**Transitioning from MODIS to VIIRS Global Water Reservoir Product**

Deep Shah^1^, Shuai Zhang^1^, Sudipta Sarkar^2,3^, Carol Davidson^2,3^, Rui Zhang^3,4^, Maosheng Zhao^2,3^, Sadashiva Devadiga^3^, Praveen Noojipady^2,3^, Miguel O. Román^5^, and Huilin Gao^1^

1. Zachry Department of Civil and Environmental Engineering, Texas A&M University, College Station, TX, USA
2. Science Systems and Applications Inc., Lanham, MD, USA
3. Terrestrial Information Systems Laboratory, NASA Goddard Space Flight Center, Greenbelt, MD, USA
4. Global Science & Technology Inc, Greenbelt, MD, USA
5. Leidos, Inc., Reston, VA, USA

**Corresponding author**: Huilin Gao (hgao@civil.tamu.edu)

**Contents of the file:**

Figures: S1-S11

Tables: S1-S4

| 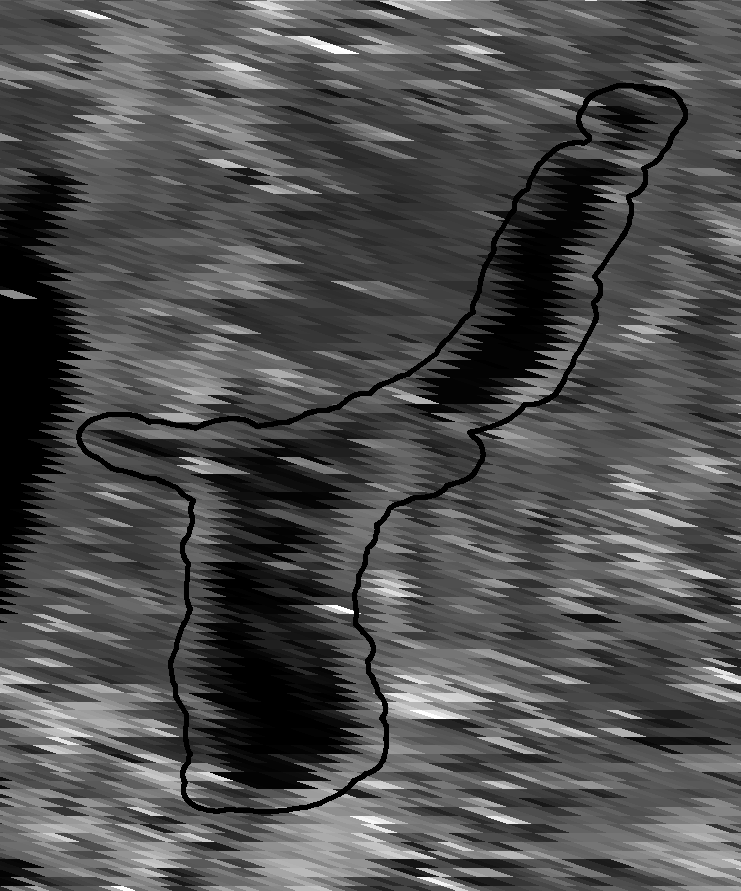 | 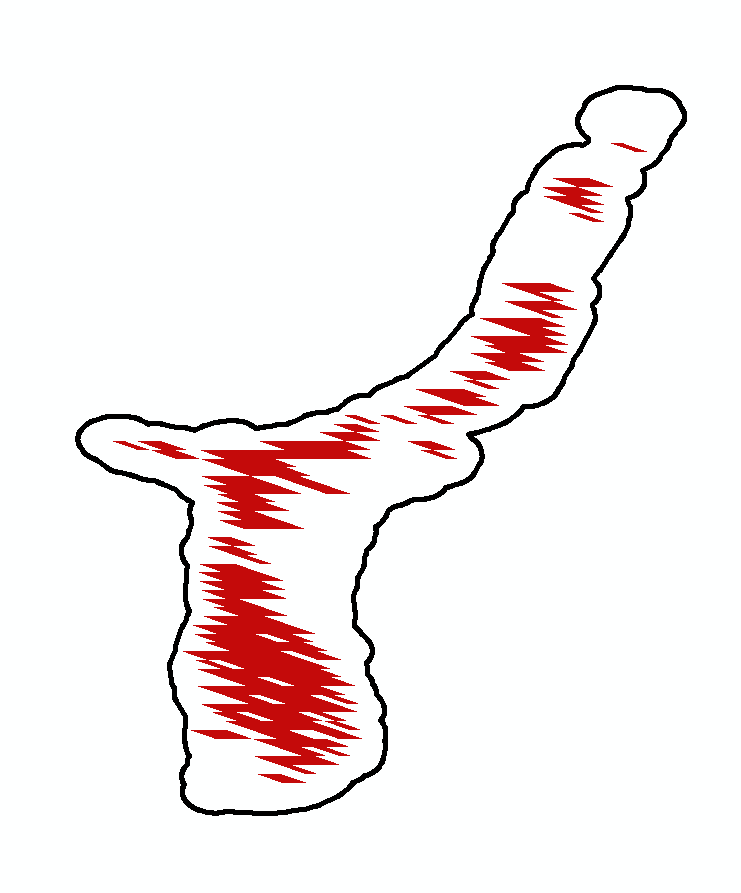 | 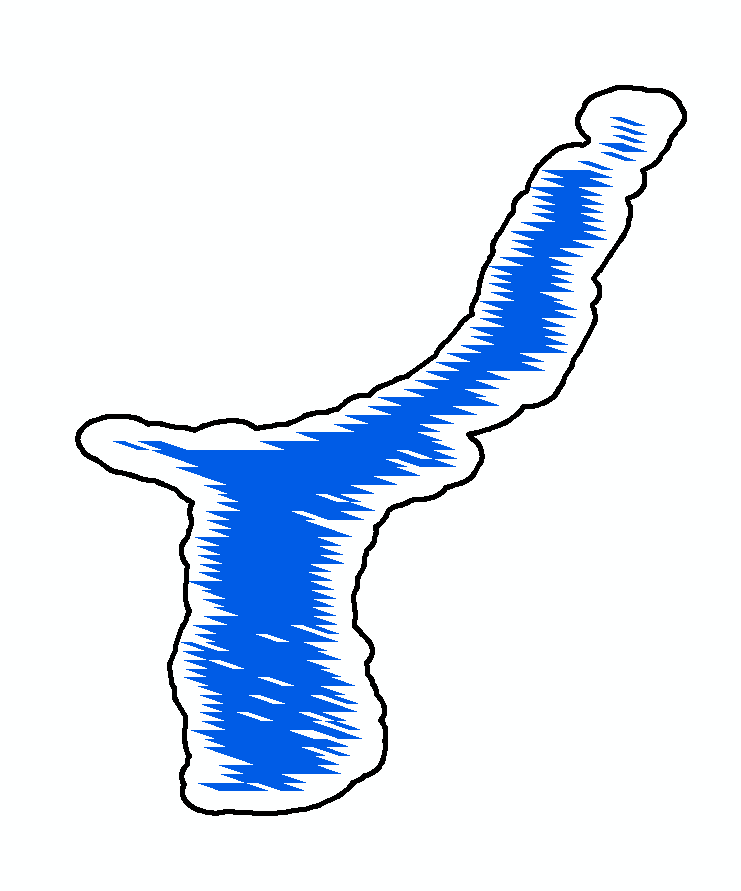 |
| --- | --- | --- |
| (a) | (b) | (c) |

**Figure S1**. The VNP image was collected on day 347 of 2021 over Lake Hawea (ID 131), New Zealand. (a) The original reflectance image; (b) the raw water from the OSTU classification; (c) the water extraction data, after improvement by the enhancement operation.


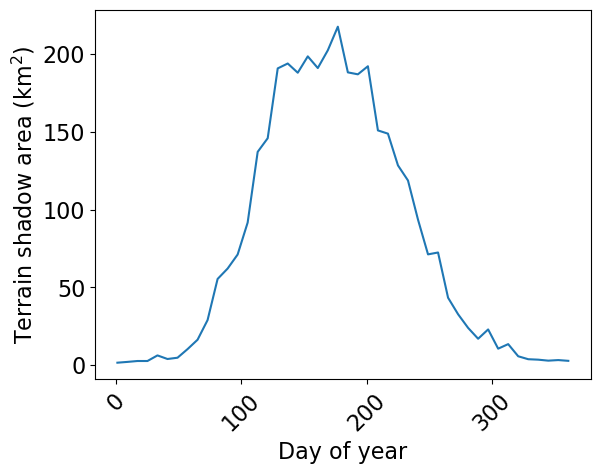


**Figure S2**. The climatology of the terrain shadow area within Lake Hawea (ID 131).

*
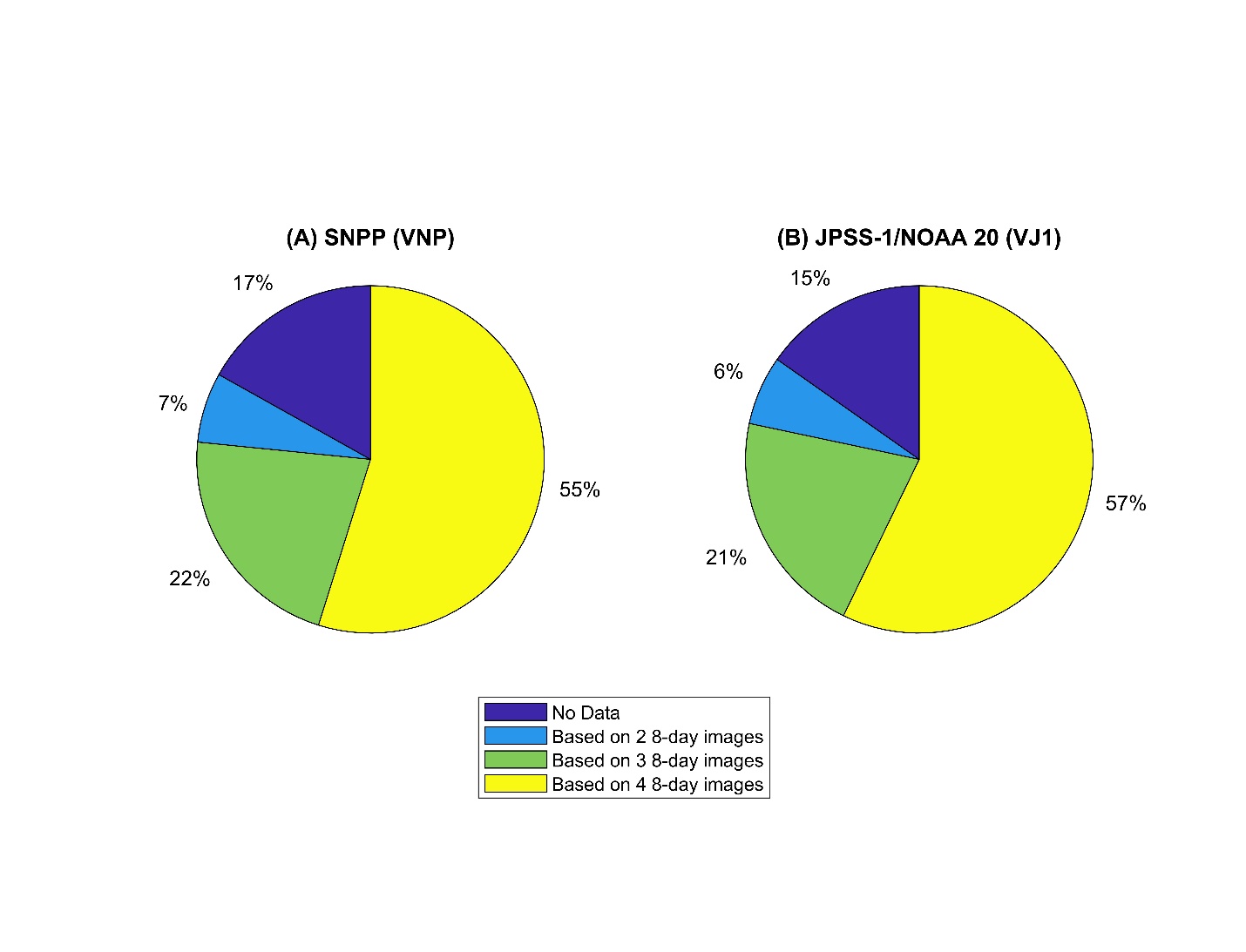
*

**Figure S3.** The percentage of the monthly data generated from different numbers of the 8-day images. (A) for SNPP (VNP), and (B) for JPSS-1/NOAA 20 (VJ1).


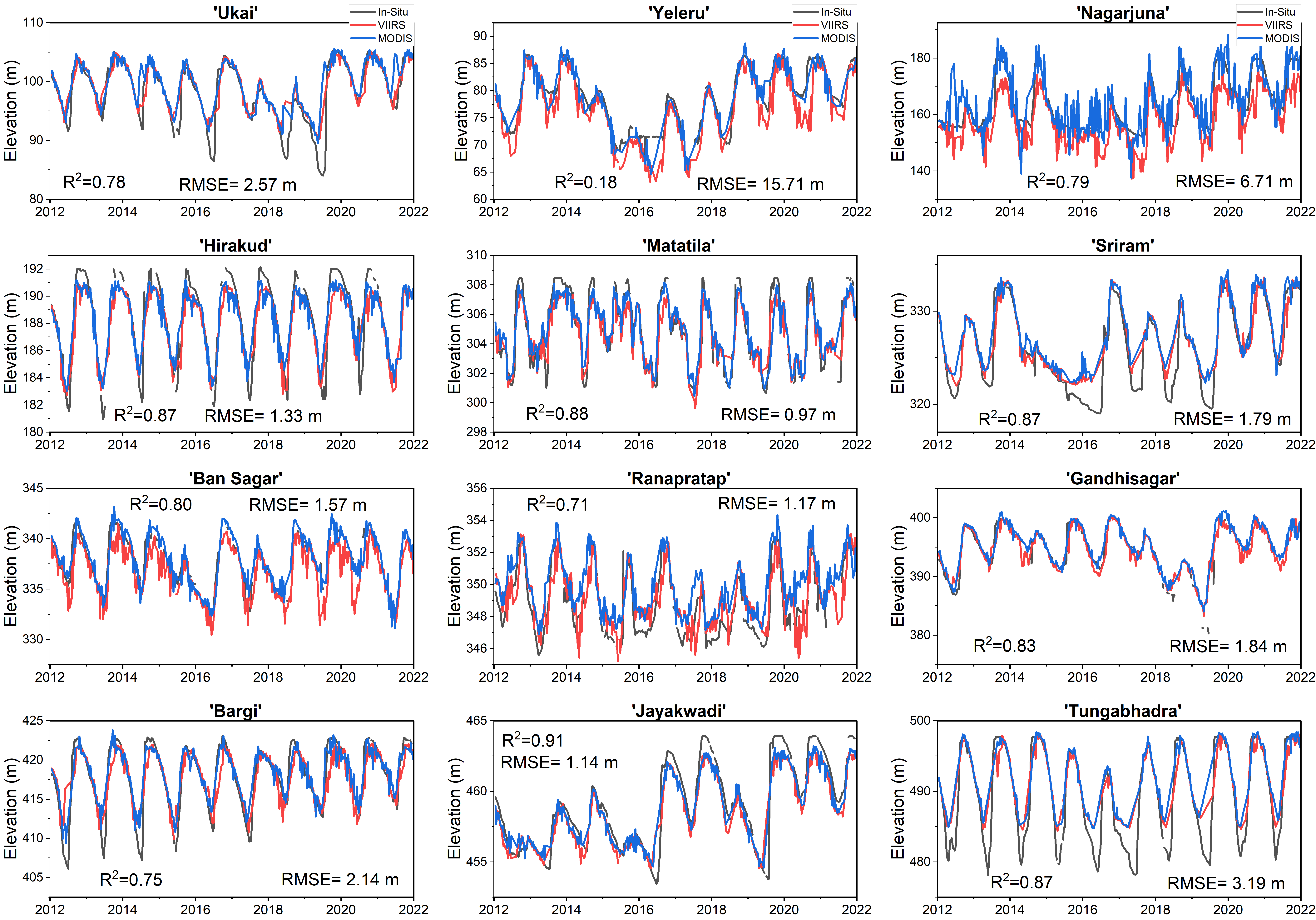


**Figure S4.** Validation of the VIIRS (VNP28C2) 8-day elevation data (red) against the in-situ (black) and MODIS (MOD28C2) elevation (blue) observations for twelve Indian reservoirs from 2012 to 2021.


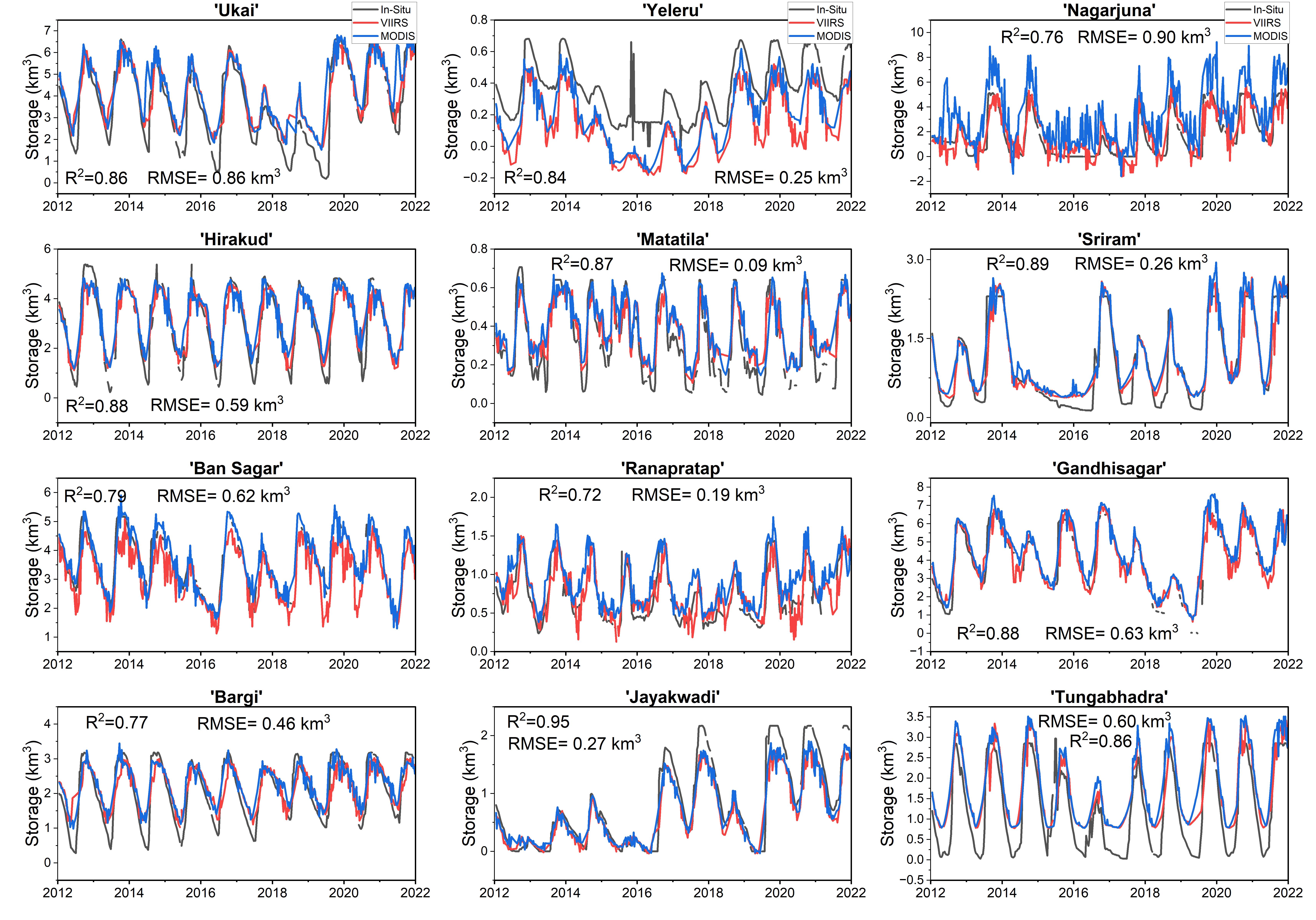


**Figure S5.** Validation of VIIRS (VNP28C2) 8-day storage data (red) against the in-situ (black) and MODIS (MOD28C2) storage (blue) observations for twelve Indian reservoirs from 2012 to 2021.


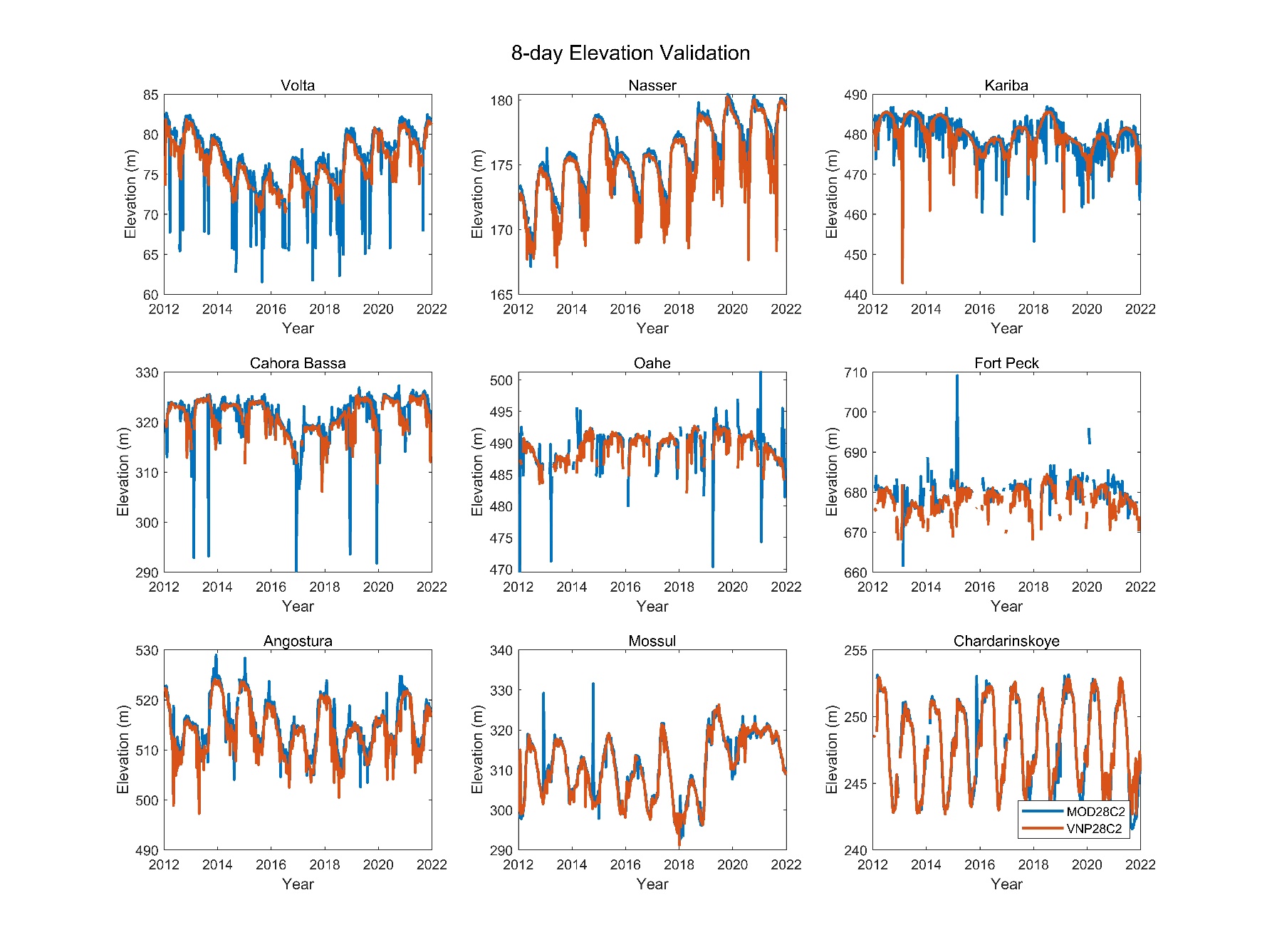


**Figure S6.** Comparison of VIIRS (VNP28C2) and MODIS (MOD28C2) 8-day raw elevation data from 2012 to 2021 at nine locations across the globe.


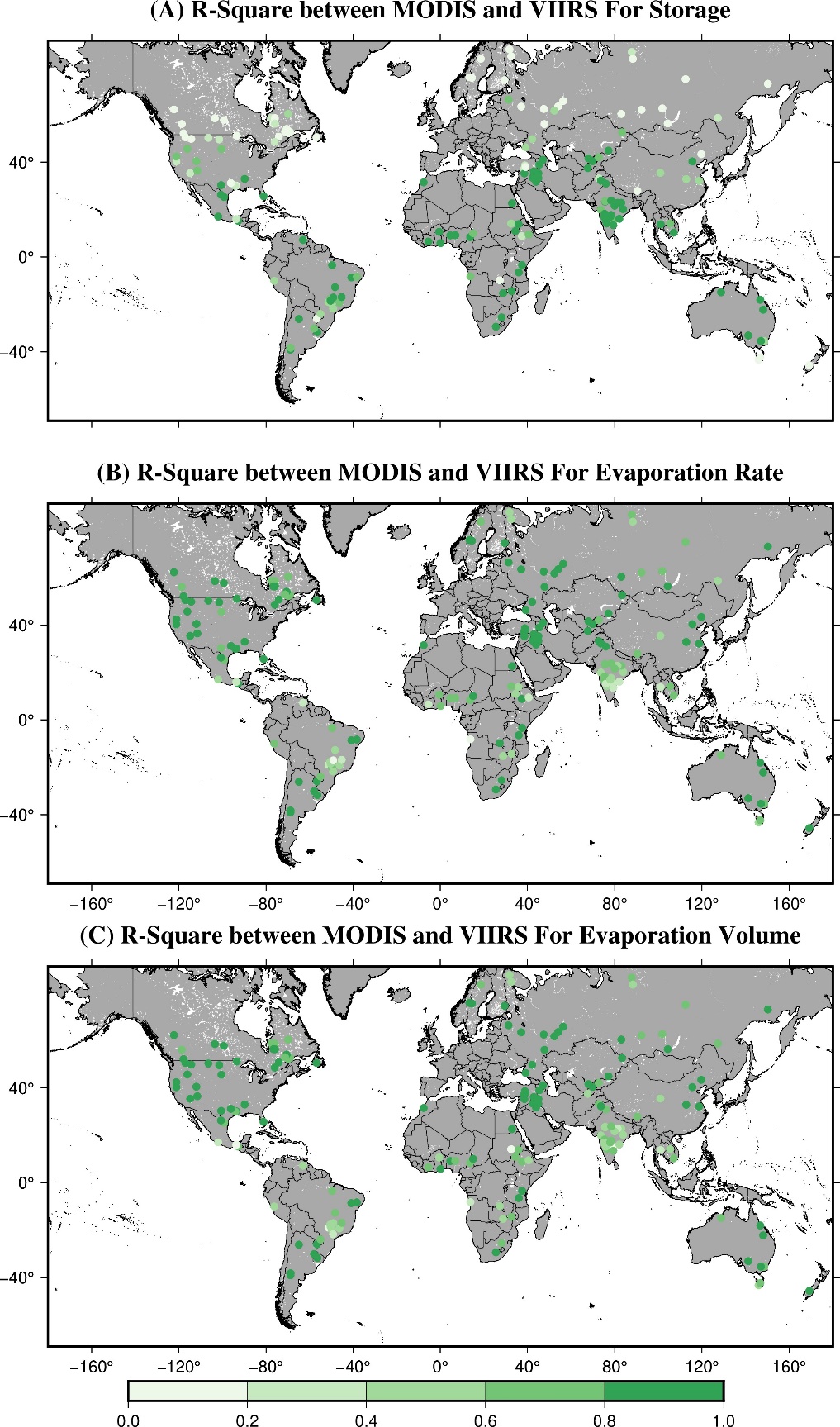


**Figure S7.** R^2^ values between the VIIRS (VNP28C3) and MODIS Terra (MOD28C3) products. (A) R^2^ of monthly storage, (B) R^2^ of evaporation rate, and (C) R^2^ of evaporation volume from 2012 to 2021 at 164 global reservoirs.

**
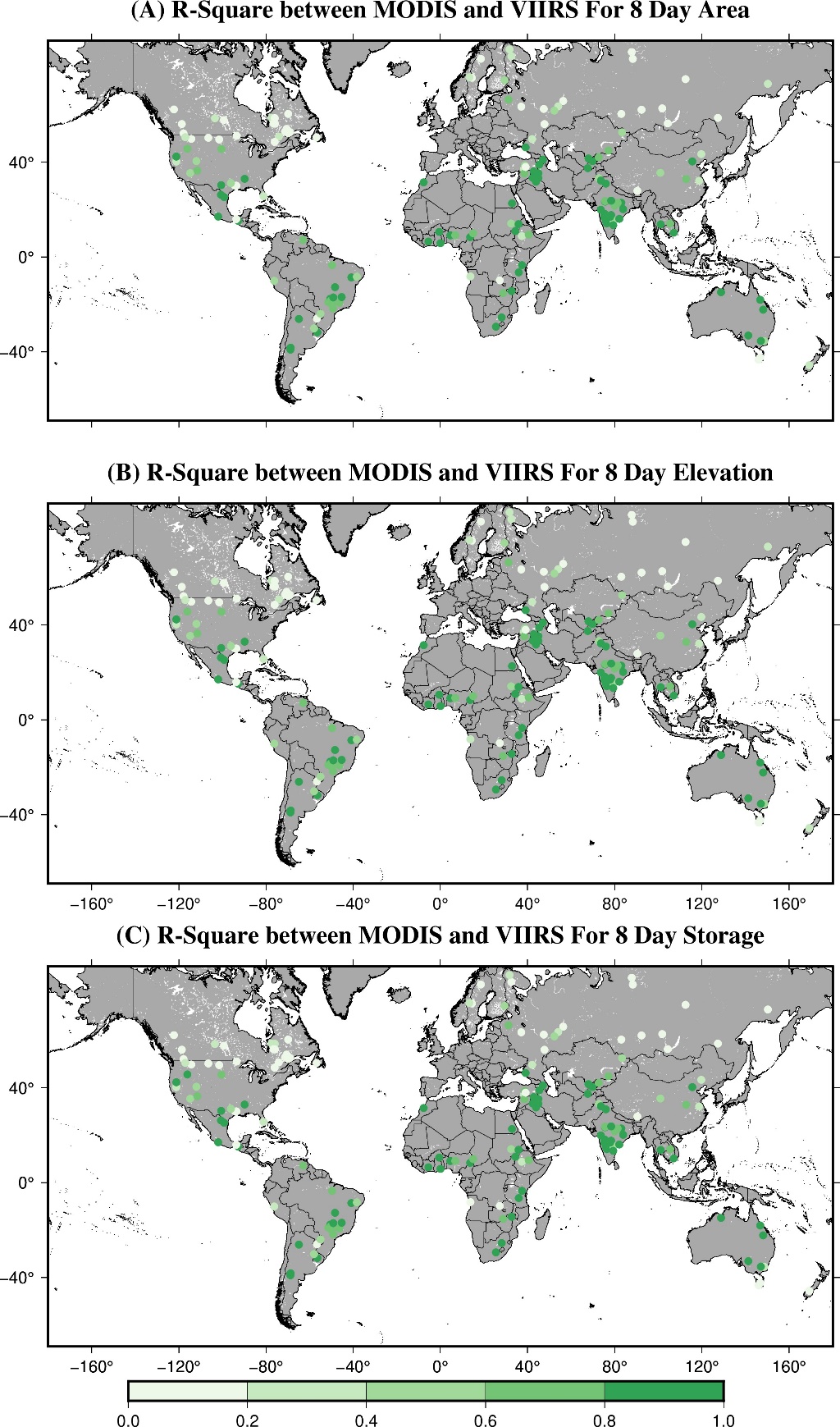
**

**Figure S8.** R^2^ values between the VIIRS (VNP28C2) and MODIS Terra (MOD28C2) products. (A) R^2^ of 8-day area, (B) R^2^ of 8-day elevation, and (C) R^2^ of 8-day storage from 2012 to 2021 at 164 global reservoirs.


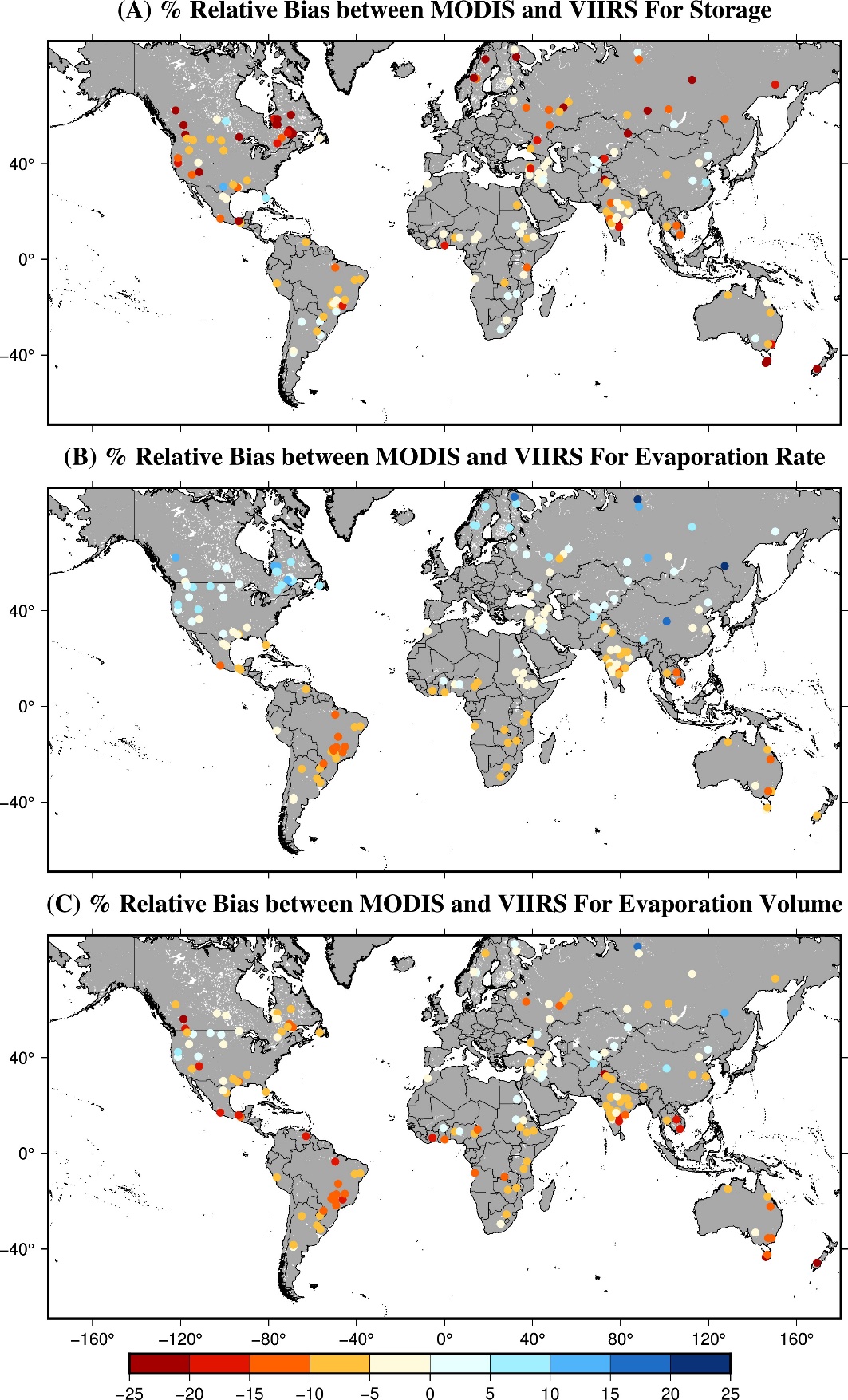


**Figure S9.** Relative Bias (RB) values (in %) between the VIIRS (VNP28C3) and MODIS Terra (MOD28C3) products. (A) % RB of monthly storage, (B) % RB of evaporation rate, and (C) % RB of evaporation volume from 2012 to 2021 at 164 global reservoirs.

**
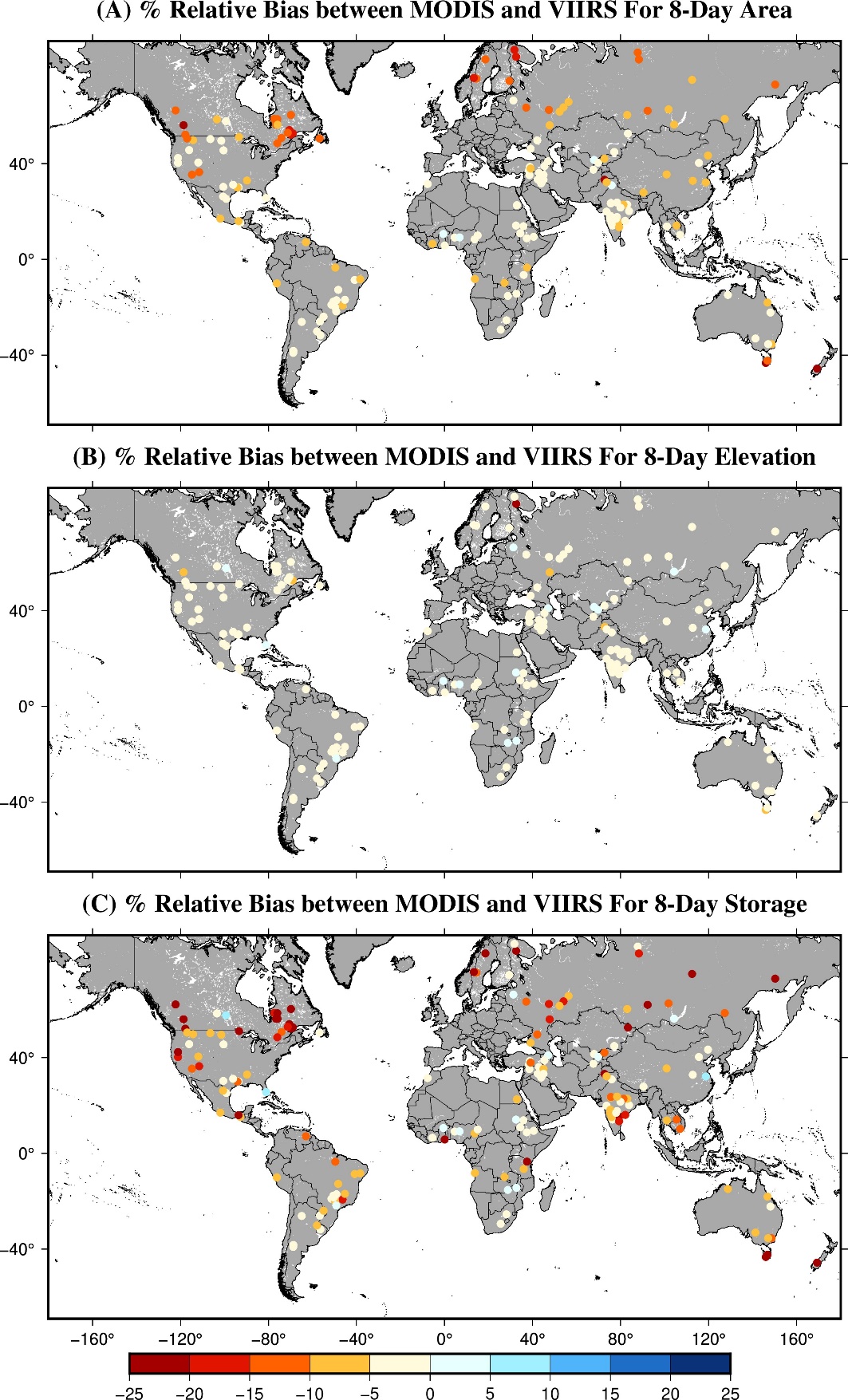
**

**Figure S10.** Relative Bias (RB) values (in %) between the VIIRS (VNP28C2) and MODIS Terra (MOD28C2) products. (A) %RB of 8-day area, (B) % RB of 8-day elevation, and (C) % RB of 8-day storage from 2012 to 2021 at 164 global reservoirs.


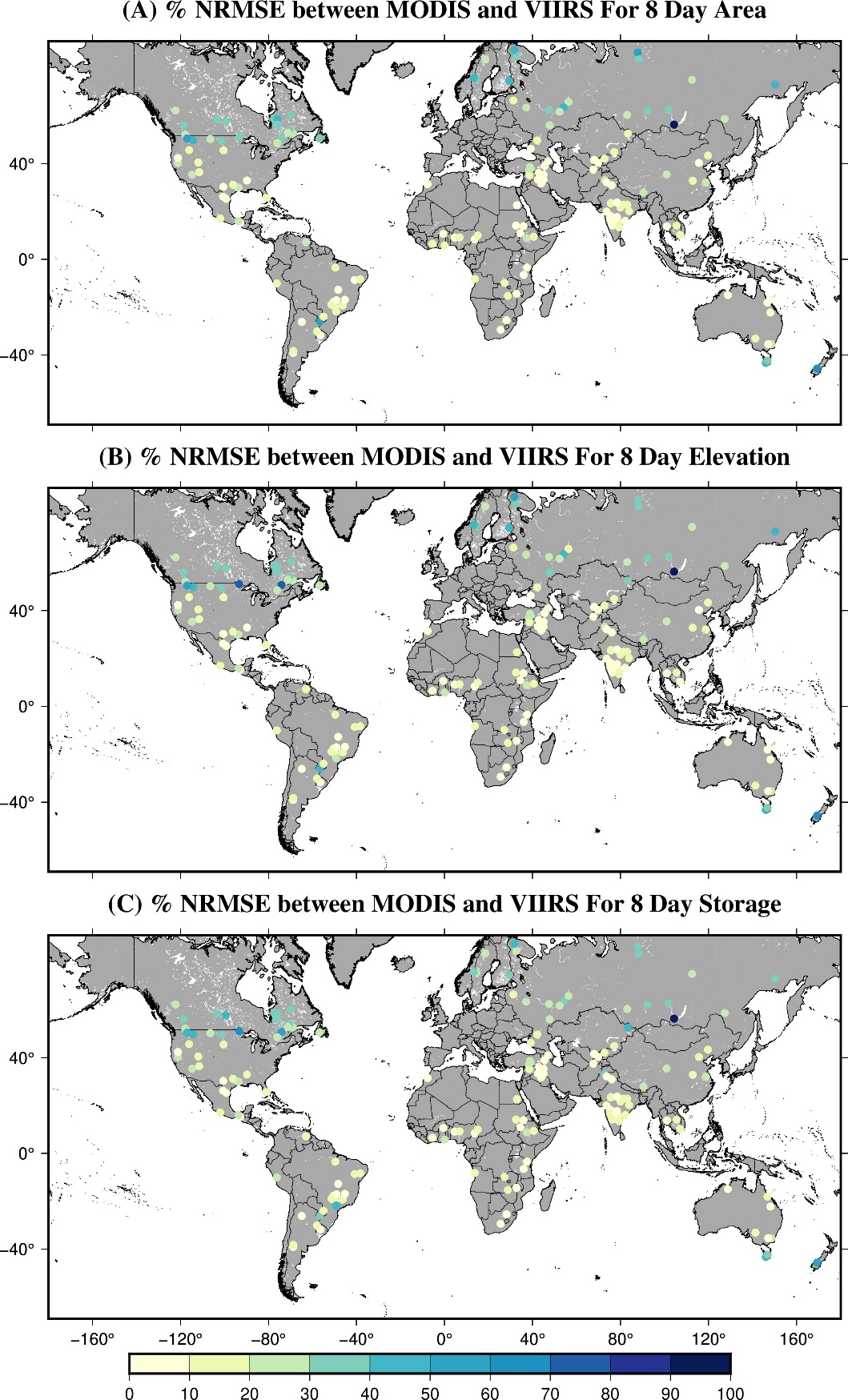


**Figure S11.** NRMSE values (in %) between the VIIRS (VNP28C2) and MODIS Terra (MOD28C2) products. (A) % NRMSE of 8-day area, (B) % NRMSE of 8-day elevation, and (C) % NRMSE of 8-day storage from 2012 to 2021 at 164 global reservoirs.

**Table S1.** Details of the 8-day and monthly MODIS and VIIRS products.

| **8-Day Product (C2)** | | |
| --- | --- | --- |
| **Instrument** | **Product Name** | **Time Span** |
| **MODIS (Terra)** | MOD28C2 | 2000 to 2021 |
| **MODIS (Aqua)** | MYD28C2 | 2002 to 2021 |
| **VIIRS (SNPP)** | VNP28C2 | 2012 to 2021 |
| **VIIRS (JPSS1)** | VJ128C2 | March 2020 to December 2021 |
| **Monthly Product (C3)** | | |
| **Instrument** | **Product Name** | **Time Span** |
| **MODIS (Terra)** | MOD28C3 | 2000 to 2021 |
| **MODIS (Aqua)** | MYD28C3 | 2002 to 2021 |
| **VIIRS (SNPP)** | VNP28C3 | 2012 to 2021 |
| **VIIRS (JPSS1)** | VJ128C3 | March 2020 to December 2021 |

**Table S2.** Summary of the input data names, sources, and purposes used in the reservoir product.

| **Input Data** | **Source** | **Purpose** |
| --- | --- | --- |
| 8-Day MODIS/VIIRS Surface Reflectance | MxD09Q1/Vyy09H1 | Area |
| Area-Elevation (A-E) relationship | GRBD | Elevation and Storage |
| 8-day MODIS/VIIRS land surface temperature | MxD11A2/Vyy21A2 | Evaporation Rate |
| Meteorological Data | GLDAS | Evaporation Rate |

**Table S3.** Summary of the differences in the MODIS and VIIRS water reservoir product algorithms.

|  | **MODIS** | **VIIRS** |
| --- | --- | --- |
| **Terrain Shadow Mask** | No | Yes |
| **Incorporation of the QA band** | No | Yes |
| **Adopt snow/ice cover fractions for quantifying evaporation volume** | No | Yes |
| **Improve the enhancement algorithm using an edge detection approach** | No (based on Zhang et al., 2014) | Yes (based on Zhao et al., 2020) |

**Table S4.** Detailed information about the 164 reservoirs and their attributes.

| ID | GRAND_ID | Hylak_id |  | Res_name | Country | Continent | a,b | storage new | area new | elevation new | Capacity_source | lon,lat |
| --- | --- | --- | --- | --- | --- | --- | --- | --- | --- | --- | --- | --- |
| 1 | 5058 | 11 |  | Baikal | Russia | Asia | 0.00447, 312.77026 | 23615.39 | 32265.61 | 456.88 | GRanD | 104.32, 52.24 |
| 2 | 3667 | 156 |  | Volta | Ghana | Africa | 0.00365, 55.58562 | 148 | 8502 | 86.65 | wikipedia | 0.06, 6.3 |
| 3 | 4478 | 152 |  | Nasser | Egypt | Africa | 0.00469, 152.81994 | 162 | 6500 | 183.28 | literature | 32.89, 23.97 |
| 4 | 4056 | 172 |  | Kariba Reservoir | Zambia | Africa | 0.01119, 424.98467 | 180 | 5400 | 485.41 | wikipedia | 28.76, -16.52 |
| 5 | 5055 | 110 |  | Bratsk Reservoir | Russia | Asia | 0.00657, 367.92163 | 169.27 | 5470 | 403.85 | wikipedia | 101.78, 56.29 |
| 6 | 4787 | 122 |  | Zaysan | Kazakhstan | Asia | 0.00465, 370.20585 | 49.8 | 5490 | 395.74 | GRanD | 83.35, 49.66 |
| 7 | 2294 | 73 |  | Guri Reservoir | Venezuela | South America | 0.0144, 217.16716 | 135 | 4250 | 278.38 | wikipedia | -63, 7.77 |
| 8 | 1995 | 43 |  | Caniapiscau Reservoir | Canada | North America | 0.01218, 488.99841 | 53.79 | 4275 | 541.08 | GRanD | -69.78, 54.85 |
| 9 | 1394 | 46 |  | Robert Bourassa Reservoir | Canada | North America | 0.0111, 143.99061 | 61.7 | 2905 | 176.24 | Hydro-Québec | -77.45, 53.79 |
| 10 | 2516 | 77 |  | Sobradinho Reservoir | Brazil | South America | 0.00571, 375.26816 | 34.1 | 3017.9 | 392.5 | GRanD | -40.82, -9.42 |
| 11 | 712 | 51 |  | Cedar | Canada | North America | 0.00217, 250.49224 | 9.64 | 2668.46 | 256.29 | GRanD | -99.29, 53.16 |
| 12 | 1396 | 47 |  | La Grande 3 Reservoir | Canada | North America | 0.02539, 195.25843 | 60 | 2451 | 257.48 | Hydro-Québec | -75.96, 53.73 |
| 13 | 2365 | 76 |  | Tucurui Reservoir | Brazil | South America | 0.01322, 40.95573 | 45.5 | 2606 | 75.4 | GRanD | -49.65, -3.83 |
| 14 | 4375 | 128 |  | Tsimlyanskoye Reservoir | Russia | Euro | 0.01177, 7.63989 | 23.86 | 2702 | 39.44 | literature | 42.11, 47.61 |
| 15 | 5834 | 115 |  | Zeyskoye Reservoir | Russia | Asia | 0.02065, 266.43675 | 68.4 | 2420 | 316.41 | wikipedia | 127.31, 53.77 |
| 16 | 5180 | 96 |  | Vilyuy Reservoir | Russia | Asia | 0.02852, 182.74156 | 35.9 | 2170 | 244.62 | wikipedia | 112.48, 63.03 |
| 17 | 4783 | 93 |  | Khantayskoye Reservoir | Russia | Asia | 0.00445, 49.76375 | 23.5 | 2221.61 | 59.64 | GRanD | 87.81, 68.16 |
| 18 | 4505 | 171 |  | Cahora Bassa Reservoir | Mozambique | Africa | 0.01542, 286.9568 | 55.8 | 2739 | 329.18 | wikipedia | 32.7, -15.58 |
| 19 | 6 | 40 |  | Williston | Canada | North America | 0.0529, 580.99344 | 39.47 | 1773 | 674.79 | literature | -122.2, 56.02 |
| 20 | 4472 | 144 |  | Buhayrat ath Tharthar | Iraq | Asia | 0.03955, -19.46261 | 85.59 | 2135.54 | 65 | literature | 43.46, 33.69 |
| 21 | 5056 | 112 |  | Krasnoyarsk Reservoir | Russia | Asia | 0.03863, 162.77316 | 73.3 | 2000 | 240.04 | wikipedia | 92.29, 55.93 |
| 22 | 4623 | 106 |  | Kama Reservoir | Russia | Euro | 0.00744, 96.07894 | 12.2 | 1915 | 110.32 | wikipedia | 56.34, 58.12 |
| 23 | 1957 | 69 |  | Okeechobee | United States of America | North America | 0.00617, -5.57499 | 3.546 | 1536.8 | 3.9 | wikipedia | -81.1, 26.94 |
| 24 | 5295 | 145 |  | Hungtze | China | Asia | 0.00749, 1.45816 | 13.5 | 2074.61 | 17 | literature | 118.73, 33.09 |
| 25 | 4474 | 146 |  | Razazah | Iraq | Asia | 0.01457, 11.06852 | 25.75 | 1621 | 34.69 | literature | 43.89, 32.7 |
| 26 | 2023 | 60 |  | Gouin Reservoir | Canada | North America | 0.00068, 402.90611 | 8.57 | 1570 | 403.98 | GRanD | -74.1, 48.36 |
| 27 | 4789 | 135 |  | Qapshaghay Bogeni Reservoir | Kazakhstan | Asia | 0.00897, 467.10974 | 28.1 | 1850 | 483.71 | GRanD | 77.1, 43.92 |
| 28 | 753 | 62 |  | Fort Berthold Reservoir | United States of America | North America | 0.02467, 528.64792 | 29.38 | 1477.4 | 565.1 | wikipedia | -101.43, 47.51 |
| 29 | 2445 | 83 |  | Aperea Reservoir | Paraguay | South America | 0.02242, 48.84199 | 21 | 1600 | 84.71 | literature | -56.63, -27.39 |
| 30 | 870 | 65 |  | Oahe | United States of America | North America | 0.02172, 462.72715 | 28.35 | 1429.57 | 493.78 | wikipedia | -100.4, 44.46 |
| 31 | 2390 | 80 |  | Ilha Solteira Reservoir | Brazil | South America | 0.03237, 290.94542 | 21.17 | 1200 | 329.78 | GRanD | -51.38, -20.37 |
| 32 | 4629 | 118 |  | Saratov Reservoir | Russia | Euro | 0.02563, -0.27741 | 12.9 | 1117.7 | 28.36 | GRanD | 47.76, 52.05 |
| 33 | 4350 | 94 |  | Imandra | Russia | Euro | 0.18726, -62.86735 | 10.8 | 1062.37 | 136.07 | GRanD | 32.55, 67.41 |
| 34 | 3640 | 155 |  | Kainji Reservoir | Nigeria | Africa | 0.03997, 93.99579 | 15 | 1071.23 | 136.81 | wikipedia | 4.61, 9.87 |
| 35 | 4785 | 113 |  | Novosibirskoye | Russia | Asia | 0.01419, 98.78019 | 8.8 | 1070 | 113.97 | wikipedia | 83, 54.84 |
| 36 | 4625 | 111 |  | Cheboksary | Russia | Euro | 0.02447, 39.29789 | 13.85 | 1080.38 | 65.73 | literature | 47.46, 56.14 |
| 37 | 4359 | 1163 |  | Ilmen | Russia | Euro | 0.0083, 9.98411 | 12 | 1120 | 19.28 | wikipedia | 31.28, 58.46 |
| 38 | 4480 | 1527 |  | Jebel Aulia Reservoir | Sudan | Africa | 0.00624, 375.01032 | 3.5 | 861.19 | 380.39 | FAO | 32.48, 15.24 |
| 39 | 1397 | 623 |  | Opinaca Reservoir | Canada | North America | 0.02118, 194.07727 | 8.5 | 1040 | 216.1 | wikipedia | -76.58, 52.21 |
| 40 | 2392 | 943 |  | Furnas | Brazil | South America | 0.0437, 720.07262 | 22.59 | 1127.07 | 769.32 | wikipedia | -46.31, -20.67 |
| 41 | 2368 | 922 |  | Serra da Mesa Reservoir | Brazil | South America | 0.03356, 410.19963 | 54.4 | 1784 | 470.07 | wikipedia | -48.3, -13.84 |
| 42 | 4624 | 1169 |  | Votkinsk Reservoir | Russia | Euro | 0.03892, 53.1356 | 9.4 | 850.82 | 86.25 | wikipedia | 54.08, 56.8 |
| 43 | 6201 | 1632 |  | Argyle Reservoir | Australia | Oceania | 0.02806, 66.43617 | 10.76 | 981.21 | 93.97 | wikipedia | 128.74, -16.12 |
| 44 | 731 | 710 |  | Rainy | Canada | North America | 0.00078, 336.08674 | 0.69 | 829.45 | 336.73 | GRanD | -93.36, 48.62 |
| 45 | 307 | 721 |  | Fort Peck | United States of America | North America | 0.04376, 643.31691 | 22.77 | 969.86 | 685.76 | wikipedia | -106.41, 48 |
| 46 | 2375 | 928 |  | Tres Marias Reservoir | Brazil | South America | 0.03553, 539.11233 | 21 | 1040 | 576.06 | wikipedia | -45.27, -18.21 |
| 47 | 2012 | 697 |  | Pipmuacan Reservoir | Canada | North America | 0.0498, 360.46403 | 13.9 | 978 | 409.16 | wikipedia | -69.77, 49.36 |
| 48 | 4679 | 1307 |  | Chardarinskoye | Kazakhstan | Asia | 0.01786, 238.24413 | 5.7 | 800.66 | 252.54 | wikipedia | 67.96, 41.25 |
| 49 | 4626 | 1175 |  | Nizhnekamsk Reservoir | Russia | Euro | 0.0138, 50.37324 | 13.8 | 1084 | 65.34 | wikipedia | 52.28, 55.7 |
| 50 | 2456 | 966 |  | Negro Reservoir | Uruguay | South America | 0.0194, 62.00777 | 8.8 | 1070 | 82.77 | wikipedia | -56.42, -32.83 |
| 51 | 2343 | 981 |  | Chocon Reservoir | Argentina | South America | 0.01519, 365.74893 | 22 | 820 | 378.2 | GRanD | -68.76, -39.27 |
| 52 | 4442 | 1348 |  | Ataturk Dam | Turkey | Asia | 0.10643, 454.25042 | 48.7 | 817 | 541.2 | GRanD | 38.32, 37.49 |
| 53 | 2513 | 915 |  | Itaparica Reservoir | Brazil | South America | 0.03337, 279.33376 | 10.7 | 781.21 | 305.4 | wikipedia | -38.31, -9.14 |
| 54 | 4464 | 1365 |  | Assad | Syria | Asia | 0.05942, 266.62629 | 11.7 | 610 | 302.87 | wikipedia | 38.55, 35.86 |
| 55 | 3650 | 1558 |  | Lagdo Reservoir | Cameroon | Africa | 0.0374, 190.15542 | 7.7 | 691.12 | 216 | FAO | 13.69, 9.06 |
| 56 | 1269 | 838 |  | Toledo Bend Reservoir | United States of America | North America | 0.02039, 39.45546 | 5.52 | 636.18 | 52.43 | wikipedia | -93.57, 31.18 |
| 57 | 6922 | 624 |  | Eastmain Reservoir | Canada | North America | 0.06785, 245.91598 | 6.94 | 602.9 | 286.82 | literature | -75.89, 52.19 |
| 58 | 2009 | 688 |  | Outardes 4 Reservoir | Canada | North America | 0.19049, 239.61011 | 24.5 | 640 | 361.53 | Hydro-Québec | -68.91, 49.71 |
| 59 | 4349 | 1036 |  | Kovdozero | Russia | Euro | 0.00193, 78.17686 | 11.52 | 745 | 79.62 | GRanD | 31.76, 68.6 |
| 60 | 2380 | 931 |  | Sao Simao Reservoir | Brazil | South America | 0.0523, 369.16877 | 12.5 | 703 | 405.94 | wikipedia | -50.5, -19.02 |
| 61 | 610 | 809 |  | Mead | United States of America | North America | 0.13619, 288.76038 | 34.07 | 659.3 | 374.6 | USBR | -114.73, 36.02 |
| 62 | 5087 | 1473 |  | Yamdrok | China | Asia | 0.01275, 4435.35521 | 14.6 | 638 | 4443.49 | literature | 90.38, 29.1 |
| 63 | 1391 | 866 |  | Angostura Reservoir | Mexico | North America | 0.08079, 478.95889 | 18.2 | 640 | 530.67 | wikipedia | -92.78, 16.4 |
| 64 | 4991 | 1524 |  | Srisailam Reservoir | India | Asia | 0.03079, 253.3044 | 8.29 | 534.05 | 269.75 | CWC | 78.9, 16.09 |
| 65 | 2455 | 964 |  | Grande Reservoir | Argentina | South America | 0.03068, 16.88963 | 5 | 592.83 | 35.08 | wikipedia | -57.94, -31.27 |
| 66 | 4843 | 1484 |  | Gandhisagar Reservoir | India | Asia | 0.03366, 379.03449 | 6.83 | 619.89 | 399.9 | CWC | 75.55, 24.7 |
| 67 | 2397 | 946 |  | Promissao Reservoir | Brazil | South America | 0.08038, 342.73167 | 7.41 | 513.39 | 384 | GRanD | -49.78, -21.3 |
| 68 | 282 | 698 |  | Arrow | Canada | North America | 0.17477, 351.0668 | 10.3 | 504.82 | 439.3 | USACE | -117.78, 49.34 |
| 69 | 2382 | 934 |  | Agua Vermelha Reservoir | Brazil | South America | 0.05626, 351.61681 | 11.03 | 563.15 | 383.3 | wikipedia | -50.35, -19.87 |
| 70 | 4898 | 1502 |  | Hirakud Reservoir | India | Asia | 0.02204, 177.26302 | 5.38 | 669.62 | 192.02 | CWC | 83.85, 21.52 |
| 71 | 3041 | 1568 |  | Kossour Reservoir | Ivory Coast | Africa | 0.03423, 169.77945 | 27.68 | 1058.2 | 206 | GRanD | -5.47, 7.03 |
| 72 | 4784 | 1058 |  | Kureiskaya | Russia | Asia | 0.04971, 67.89284 | 9.96 | 558 | 95.63 | literature | 88.29, 66.95 |
| 73 | 3071 | 1104 |  | Storsjon | Sweden | Euro | 0.00422, 291.0872 | 0.5 | 484.6 | 293.13 | GRanD | 14.47, 63.3 |
| 74 | 316 | 730 |  | Flathead Lake | United States of America | North America | 0.13239, 816.09051 | 23.2 | 510 | 883.61 | wikipedia | -114.23, 47.68 |
| 75 | 2004 | 661 |  | Kempt | Canada | North America | 0.03312, 478.60112 | 2.22 | 470.44 | 494.18 | GRanD | -70.53, 50.66 |
| 76 | 6700 | 1123 |  | Kolyma dam | Russia | Asia | 0.13658, 390.9085 | 15.08 | 454.6 | 453 | wikipedia | 150.23, 62.05 |
| 77 | 4501 | 1612 |  | Mtera Reservoir | United Republic of Tanzania | Africa | 0.02183, 688.04662 | 3.2 | 478.83 | 698.5 | literature | 35.98, -7.14 |
| 78 | 4686 | 1320 |  | Kayrakkumskoye | Tajikistan | Asia | 0.02143, 335.23897 | 4.2 | 513 | 346.23 | wikipedia | 69.82, 40.28 |
| 79 | 250 | 628 |  | Kinbasket | Canada | North America | 0.31717, 622.76738 | 24.76 | 430 | 759.15 | wikipedia | -118.57, 52.08 |
| 80 | 4634 | 1313 |  | Mingechaurskoye | Azerbaijan | Asia | 0.07215, 42.01887 | 15.73 | 567.97 | 83 | wikipedia | 47.03, 40.8 |
| 81 | 2431 | 956 |  | Lago del Río Yguazú | Paraguay | South America | 0.04517, 203.13232 | 8.47 | 620 | 231.14 | wikipedia | -54.97, -25.37 |
| 82 | 4858 | 1487 |  | Govind Ballabah Pant | India | Asia | 0.06208, 241.75327 | 5.65 | 426.36 | 268.22 | CWC | 83, 24.2 |
| 83 | 4422 | 1332 |  | Keban Baraji | Turkey | Asia | 0.11302, 772.50564 | 30.6 | 675 | 848.79 | wikipedia | 38.76, 38.81 |
| 84 | 2340 | 978 |  | Los Barreales | Argentina | South America | 0.30759, 290.07305 | 27.7 | 413 | 417.11 | literature | -68.69, -38.58 |
| 85 | 4859 | 1488 |  | Bansagar Lake | India | Asia | 0.05088, 317.64432 | 5.17 | 471.6 | 341.64 | CWC | 81.29, 24.19 |
| 86 | 1275 | 839 |  | Sam Rayburn Reservoir | United States of America | North America | 0.0355, 35.65711 | 3.55 | 455.64 | 50.11 | TWDB | -94.11, 31.07 |
| 87 | 2414 | 953 |  | Barra Bonita | Brazil | South America | 0.00228, 565.24837 | 7.01 | 542 | 566.48 | GRanD | -49.23, -23.21 |
| 88 | 4739 | 1504 |  | Ukal | India | Asia | 0.04229, 83.59772 | 6.62 | 509.85 | 105.16 | CWC | 73.6, 21.26 |
| 89 | 479 | 788 |  | Utah Lake | United States of America | North America | 0.02307, 1359.51211 | 1.07 | 380 | 1368.28 | wikipedia | -111.89, 40.36 |
| 90 | 305 | 719 |  | Pend Oreille Lake | United States of America | North America | 0.22845, 541.65792 | 54.2 | 381.47 | 628.8 | wikipedia | -117, 48.18 |
| 91 | 4994 | 1526 |  | Tungabhadra | India | Asia | 0.04122, 483.33699 | 3.28 | 349.42 | 497.74 | CWC | 76.33, 15.27 |
| 92 | 4461 | 1355 |  | Mosul Dam Lake | Iraq | Asia | 0.16032, 273.38375 | 11.1 | 353.16 | 330 | wikipedia | 42.83, 36.63 |
| 93 | 4470 | 1392 |  | Habbaniyah | Iraq | Asia | 0.07125, 114.61642 | 8.2 | 418.4 | 144.43 | literature | 42.35, 34.21 |
| 94 | 4946 | 1509 |  | Sriramsagar Reservoir | India | Asia | 0.04005, 319.94975 | 2.3 | 314.38 | 332.54 | CWC | 78.34, 18.97 |
| 95 | 2376 | 929 |  | Lago das Brisas | Brazil | South America | 0.08818, 471.03368 | 17 | 559.6 | 520.38 | wikipedia | -49.1, -18.41 |
| 96 | 2356 | 720 |  | Meelpaeg | Canada | North America | 0.0041, 269.35893 | 2.16 | 314.9 | 270.65 | GRanD | -56.78, 48.17 |
| 97 | 4260 | 1678 |  | Hendrik Verwoerd | South Africa | Africa | 0.06907, 1236.10289 | 5.34 | 374 | 1261.93 | wikipedia | 25.5, -30.62 |
| 98 | 1387 | 864 |  | Malpaso | Mexico | North America | 0.30032, 89.06386 | 9.17 | 309.45 | 182 | literature | -93.6, 17.18 |
| 99 | 1379 | 861 |  | Inhernillo | Mexico | North America | 0.14118, 116.65544 | 12 | 400 | 173.13 | wikipedia | -101.89, 18.27 |
| 100 | 4184 | 1657 |  | Vaaldam | South Africa | Africa | 0.0358, 1472.81742 | 2.61 | 320 | 1484.27 | wikipedia | 28.12, -26.88 |
| 101 | 5062 | 1358 |  | Longyangxia | China | Asia | 0.18321, 2518.97907 | 24.7 | 383 | 2589.15 | wikipedia | 100.92, 36.12 |
| 102 | 3727 | 1111 |  | Hoytiainen | Finland | Euro | 0.0064, 86.17122 | 2.39 | 293 | 88.05 | GRanD | 29.48, 62.83 |
| 103 | 1423 | 741 |  | Baskatong | Canada | North America | 0.05663, 207.28526 | 2.63 | 280 | 223.14 | GRanD | -75.98, 46.72 |
| 104 | 5803 | 1549 |  | Tri An Lake | Vietnam | Asia | 0.07216, 39.48203 | 2.76 | 323 | 62.79 | wikipedia | 107.04, 11.11 |
| 105 | 2007 | 680 |  | Peribonka | Canada | North America | 0.10611, 411.5385 | 5.18 | 270.72 | 440.26 | GRanD | -71.25, 49.9 |
| 106 | 4942 | 1507 |  | Jayakwadi | India | Asia | 0.03201, 451.67121 | 2.17 | 382.39 | 463.91 | CWC | 75.37, 19.49 |
| 107 | 3638 | 1554 |  | Shiroro | Nigeria | Africa | 0.08602, 350.89662 | 7 | 312 | 377.73 | FAO | 6.84, 9.97 |
| 108 | 4379 | 1289 |  | Tshchikskoye | Russia | Euro | 0.06161, 16.03972 | 3.05 | 286.28 | 33.68 | FAO | 39.12, 44.99 |
| 109 | 710 | 589 |  | Tobin | Canada | North America | 0.00897, 311.22766 | 2.2 | 263.86 | 313.59 | GRanD | -103.4, 53.66 |
| 110 | 5796 | 1528 |  | Noi | Thailand | Asia | 0.05709, 129.50217 | 1.97 | 288 | 145.94 | wikipedia | 105.43, 15.21 |
| 111 | 4483 | 1543 |  | Roseires Reservoir | Sudan | Africa | 0.02506, 475.84407 | 7.4 | 450 | 487.12 | wikipedia | 34.39, 11.8 |
| 112 | 4675 | 1306 |  | Toktogul'skoye | Kyrgyzstan | Asia | 0.55471, 743.53409 | 19.5 | 284.3 | 901.24 | wikipedia | 72.65, 41.68 |
| 113 | 6698 | 1700 |  | Gordon | Australia | Oceania | 0.37007, 208.53588 | 12.4 | 278 | 311.42 | wikipedia | 145.98, -42.73 |
| 114 | 4964 | 1513 |  | Ujani | India | Asia | 0.05453, 482.16622 | 1.52 | 268.91 | 496.83 | CWC | 75.12, 18.07 |
| 115 | 2312 | 959 |  | Hondo | Argentina | South America | 0.02922, 266.72004 | 1.74 | 330 | 276.36 | WLDB | -64.89, -27.52 |
| 116 | 4362 | 1171 |  | Ivankovo Reservoir | Russia | Euro | 0.01794, 119.50914 | 1.17 | 220.57 | 123.47 | GRanD | 37.12, 56.73 |
| 117 | 4702 | 1398 |  | Tarbela | Pakistan | Asia | 0.52839, 351.45663 | 13.69 | 250 | 483.55 | wikipedia | 72.69, 34.09 |
| 118 | 4985 | 1519 |  | Nagarjuna | India | Asia | 0.29044, 100.77784 | 6.84 | 272.18 | 179.83 | wikipedia | 79.31, 16.57 |
| 119 | 3070 | 1102 |  | Kallsjon | Sweden | Euro | 0.02782, 387.52135 | 0.45 | 189.74 | 392.8 | GRanD | 13.34, 63.43 |
| 120 | 4431 | 1337 |  | Karakaya | Turkey | Asia | 0.22073, 631.76449 | 9.5 | 298 | 697.54 | wikipedia | 39.14, 38.23 |
| 121 | 4792 | 1423 |  | Beas | India | Asia | 0.20473, 371.49329 | 6.16 | 254.85 | 423.67 | CWC | 75.95, 31.97 |
| 122 | 4047 | 1622 |  | Tshangalele | Democratic Republic of the Congo | Africa | 0.03102, 1119.03312 | 1.267 | 225.65 | 1126.03 | GRanD | 27.24, -10.75 |
| 123 | 4485 | 1555 |  | Finchaa | Ethiopia | Africa | 0.01891, 2216.55235 | 0.65 | 196.13 | 2220.26 | FAO | 37.36, 9.56 |
| 124 | 4989 | 1521 |  | Almatti | India | Asia | 0.05275, 504.12335 | 3.11 | 293.42 | 519.6 | CWC | 75.89, 16.33 |
| 125 | 4707 | 1408 |  | Mangla | Pakistan | Asia | 0.20109, 320.1312 | 9.12 | 251 | 370.6 | wikipedia | 73.64, 33.15 |
| 126 | 4836 | 1481 |  | Rana Pratap | India | Asia | 0.1391, 324.74 | 1.44 | 197.66 | 352.81 | CWC | 75.58, 24.92 |
| 127 | 3014 | 1545 |  | Bagre | Burkina Faso | Africa | 0.05719, 223.53693 | 1.7 | 255 | 238.12 | literature | -0.55, 11.47 |
| 128 | 1991 | 916 |  | Junin | Peru | South America | 0.02312, 4079.83703 | 1.08 | 206.71 | 4084.62 | WLDB | -76.19, -10.98 |
| 129 | 4881 | 1496 |  | Bargi Dam Reservoir | India | Asia | 0.08518, 401.51078 | 3.18 | 236.24 | 422.76 | CWC | 79.93, 22.95 |
| 130 | 6686 | 1699 |  | Great Lake | Australia | Oceania | 0.40346, 969.53157 | 3.36 | 176 | 1040.54 | GRanD | 146.73, -41.98 |
| 131 | 6800 | 1704 |  | Hawea | New Zealand | Oceania | 0.14631, 323.54085 | 2.18 | 150 | 345.49 | GRanD | 169.25, -44.61 |
| 132 | 3676 | 1619 |  | Albufeira da Quiminha | Angola | Africa | 0.13121, 34.99206 | 1.56 | 129.05 | 51.93 | GRanD | 13.79, -8.96 |
| 133 | 6629 | 1695 |  | Eucumbene | Australia | Oceania | 0.46484, 1097.64507 | 4.8 | 145.42 | 1165.24 | wikipedia | 148.62, -36.13 |
| 134 | 1320 | 855 |  | Falcon Reservoir | United States of America | North America | 0.06972, 71.73912 | 3.88 | 311.84 | 93.48 | TWDB | -99.17, 26.56 |
| 135 | 597 | 802 |  | Lake Powell | United States of America | North America | 0.1406, 1047.2 | 30 | 609.38 | 1127.76 | wikipedia | -111.49, 36.94 |
| 136 | 4463 | 1362 |  | Dukan | Iraq | Asia | 0.18893, 462.6788 | 6.97 | 270 | 513.69 | wikipedia | 44.96, 35.96 |
| 137 | 1230 | 835 |  | Cedar Creek Reservoir | United States of America | North America | 0.09423, 85.91971 | 0.8 | 133.03 | 98.15 | TWDB | -96.07, 32.18 |
| 138 | 4041 | 1551 |  | Lake Maga | Cameroon | Africa | 0.01933, 309.62551 | 0.68 | 148.72 | 312.5 | literature | 15.05, 10.83 |
| 139 | 5157 | 1530 |  | Pasak Chonlasit | Thailand | Asia | 0.05295, 33.58769 | 0.79 | 158.87 | 42 | literature | 101.08, 14.85 |
| 140 | 6594 | 1650 |  | Fairbairn | Australia | Oceania | 0.13, 186.48395 | 2.29 | 179.43 | 209.81 | wikipedia | 148.06, -23.65 |
| 141 | 6628 | 1694 |  | Hume | Australia | Oceania | 0.15399, 161.81633 | 3.04 | 201.9 | 192 | wikipedia | 147.03, -36.11 |
| 142 | 4500 | 1605 |  | Kikuletwa | United Republic of Tanzania | Africa | 0.1, 677.01366 | 0.6 | 126.33 | 689.65 | wikipedia | 37.47, -3.82 |
| 143 | 4958 | 1511 |  | Nizam sagar | India | Asia | 0.0893, 419.95709 | 0.5 | 92.75 | 428.24 | CWC | 77.93, 18.2 |
| 144 | 6606 | 1690 |  | Victoria | Australia | Oceania | 0.16558, 7.52685 | 0.68 | 122 | 27.73 | GRanD | 141.28, -34.04 |
| 145 | 1869 | 826 |  | Grenada Lake | United States of America | North America | 0.12614, 49.34905 | 1.54 | 128.29 | 65.53 | Lakes Online | -89.77, 33.82 |
| 146 | 138 | 790 |  | Canyon | United States of America | North America | 0.68749, 1300.93547 | 1.61 | 108.39 | 1373.12 | wikipedia | -121.09, 40.18 |
| 147 | 4638 | 1329 |  | Aras Dam Lake | Azerbaijan | Asia | 0.11845, 762.76554 | 1.35 | 145 | 779.94 | wikipedia | 45.4, 39.09 |
| 148 | 4481 | 1529 |  | Khashm el-Girba | Sudan | Africa | 0.09342, 463.08227 | 1.3 | 125 | 474.76 | wikipedia | 35.9, 14.93 |
| 149 | 370 | 8978 |  | Lake Cascade | United States of America | North America | 0.16232, 1455.02068 | 0.85 | 101.98 | 1471.57 | wikipedia | -116.05, 44.52 |
| 150 | 3695 | 11663 |  | Seitevare | Sweden | Euro | 0.62918, 419.1852 | 1.68 | 81 | 470.15 | GRanD | 18.57, 66.97 |
| 151 | 4484 | 15827 |  | Yardi | Ethiopia | Africa | 0.33044, 533.59442 | 2.32 | 104.87 | 568.25 | GRanD | 40.54, 10.23 |
| 152 | 119 | 9138 |  | Clear Lake Reservoir | United States of America | North America | 0.19968, 1345.80215 | 0.65 | 100.36 | 1365.84 | wikipedia | -121.08, 41.93 |
| 153 | 5196 | 14551 |  | Guanting Shuiku | China | Asia | 0.10764, 465.09336 | 4.16 | 130 | 479.09 | GRanD | 115.6, 40.23 |
| 154 | 2953 | 15102 |  | Barrage Al Massira | Morocco | Africa | 0.33916, 241.40761 | 2.76 | 80 | 268.54 | wikipedia | -7.64, 32.47 |
| 155 | 1319 | 9634 |  | Venustiano Carranza | Mexico | North America | 0.09456, 252.29236 | 1.31 | 150.56 | 266.53 | literature | -100.62, 27.51 |
| 156 | 4471 | 15001 |  | Lake Hamrin | Iraq | Asia | 0.11963, 80.22516 | 4.61 | 228 | 107.5 | literature | 44.97, 34.12 |
| 157 | 4826 | 15490 |  | Matatila | India | Asia | 0.10028, 297.22136 | 0.71 | 112.07 | 308.46 | CWC | 78.37, 25.1 |
| 158 | 1263 | 9503 |  | Twin Buttes | United States of America | North America | 0.49517, 576.78181 | 0.23 | 29.47 | 591.37 | TWDB | -100.52, 31.37 |
| 159 | 4997 | 15748 |  | Somasila | India | Asia | 0.17144, 74.32045 | 1.99 | 153.17 | 100.58 | CWC | 79.3, 14.49 |
| 160 | 5183 | 14389 |  | Hongshan Reservoir | China | Asia | 0.23268, 422.07692 | 2.56 | 66.9 | 437.64 | GRanD | 119.7, 42.75 |
| 161 | 6583 | 16242 |  | Lake Ross | Australia | Oceania | 0.11178, 32.60497 | 0.417 | 82 | 41.77 | wikipedia | 146.74, -19.41 |
| 162 | 4978 | 15697 |  | Yeleru Reservoir | India | Asia | 0.58856, 57.51076 | 0.51 | 49.36 | 86.56 | CWC | 82.08, 17.3 |
| 163 | 4696 | 14730 |  | South Surkhan Reservoir | Uzbekistan | Asia | 0.33795, 397.79868 | 0.8 | 40.26 | 411.41 | GRanD | 67.63, 37.83 |
| 164 | 5287 | 15019 |  | Zhaopingtai Reservoir | China | Asia | 0.35804, 157.62551 | 0.71 | 46.5 | 174.27 | GRanD | 112.77, 33.73 |
